# Selective targeting of cortactin tandem repeat acetylation by human lysine deacetylases

**DOI:** 10.1111/febs.70430

**Published:** 2026-02-04

**Authors:** Jan Komarek, Miroslava Vosahlikova, Zsofia Kutil, Zora Novakova, Julia Kudlacova, Ruzena Tuckova, Marat Meleshin, Barbora Havlinova, Pavlina Jaklova, Jana Ptackova, Cordelia Schiene‐Fischer, Mike Schutkowski, Cyril Barinka

**Affiliations:** ^1^ Institute of Biotechnology of the Czech Academy of Sciences, BIOCEV Vestec Czech Republic; ^2^ Department of Enzymology Charles Tanford Protein Center, Institute of Biochemistry and Biotechnology, Martin‐Luther‐University Halle‐Wittenberg Halle/Saale Germany; ^3^ Present address: National Centre for Biomolecular Research, Faculty of Science, Czech Republic, and Central European Institute of Technology (CEITEC), Masaryk University Brno Czech Republic

**Keywords:** acetylation, cortactin, genetic code expansion, histone deacetylase (HDAC), sirtuin, substrate specificity

## Abstract

Lysine acetylation within the tandem repeat region of cortactin (CTTN) regulates its actin‐binding function and has been linked to cancer cell migration and neuronal development. While several lysine deacetylases (KDACs) have been implicated in modulating CTTN acetylation in cells, their site specificity and direct enzymatic roles remain poorly defined. Here, we use genetic code expansion to generate seven site‐specifically acetylated CTTN variants and assess their deacetylation by human KDACs in a fully reconstituted *in vitro* system. Our results identify HDAC6 as the primary CTTN deacetylase, acting via its second catalytic domain (DD2), and demonstrate that SIRT1 and SIRT2 also directly deacetylate CTTN at overlapping sites in an NAD^+^‐dependent manner. In contrast, other zinc‐dependent HDACs, including HDAC8, displayed negligible or very weak activity on full‐length CTTN. These findings provide new mechanistic insight into KDAC substrate preferences and highlight the value of biochemical reconstitution for dissecting complex acetylation networks.

## Graphical abstract

Cortactin function is regulated by acetylation at several lysine residues within its tandem repeat region. Using genetic code expansion to generate cortactin variants containing precisely defined acetylation marks, we show that HDAC6 is the primary enzyme removing these modifications, with SIRT1 and SIRT2 also acting at selected sites but with lower efficiency.

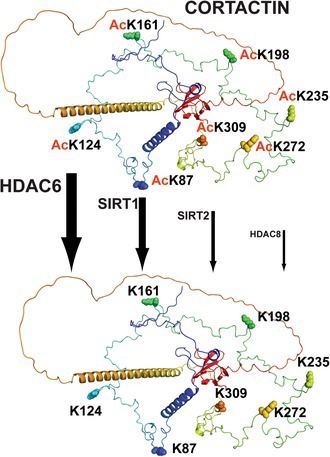

**Abbreviations**
Abz: aminobenzoic acid
AcK: N^ε^‐acetyl‐lysine
AcK‐CTTN: acetylated cortactin
CBB: Coomassie brilliant blue
CTTN: cortactin
HAT/KAT: histone/lysine acetyltransferase
HDAC/KDAC: histone/lysine deacetylase
HRP: horse radish peroxidase
IPTG: isopropyl β‐d‐thiogalactopyranoside
NAD^+^
: nicotinamide adenine dinucleotide
NAM: nicotinamide
SIRT: sirtuin
TCEP: Tris(2‐carboxyethyl)phosphine
UAA: unnatural amino acid

## Introduction

Lysine acetylation is a dynamic and evolutionarily conserved posttranslational modification that regulates a wide array of cellular processes, including gene expression, cell cycle progression, DNA repair, cytoskeleton remodeling, and autophagy [1, 2, 3]. It is reversible and tightly controlled by the opposing activities of lysine/histone acetyltransferases (KATs/HATs) and lysine/histone deacetylases (KDACs/HDACs). Human KDACs are classified into four classes: class I (HDAC1, 2, 3, 8), class II (IIa: HDAC4, 5, 7, 9; IIb: HDAC6, 10), class III (sirtuins: SIRT1–7), and class IV (HDAC11). While classical HDACs (classes I, II, IV) require Zn^2+^ for catalysis, sirtuins are NAD^+^‐dependent. KDACs deacetylate both histone and non‐histone substrates including tubulin, p53, and Hsp90 [4, 5, 6, 7, 8, 9, 10, 11, 12, 13, 14, 15, 16, 17]. Given their broad range of physiological substrates, dysregulation of the (de)acetylation machinery has been linked to numerous diseases, including cancer, neurodegeneration, and metabolic and inflammatory disorders [18, 19, 20, 21].

Cortactin (CTTN) is an actin‐binding protein that promotes filament branching and polymerization. Originally identified as a substrate of Src kinase [22, 23], cortactin is enriched in dynamic actin structures like lamellipodia and membrane ruffles [24, 25, 26]. It is often overexpressed in cancers, promoting cell migration and metastasis [27, 28]. Historically, several structural/functional domains and/or motifs have been assigned to CTTN, including the N‐terminal acidic domain (NTA) that binds Arp2/3 [25, 29]; a central tandem repeat domain comprising 6.5 copies of a unique, 37 amino acid sequence critical for F‐actin binding [25, 26]; and a C‐terminal Src homology 3 domain (SH3) that mediates protein interactions [30]. Additional regions include an α‐helical segment of unknown function and a proline‐rich domain as a primary phosphorylation site [31].

CTTN is extensively acetylated *in vivo*, particularly within its central, lysine‐rich tandem repeat region. Several KATs—including p300/CBF‐associated factor (PCAF), p300, α‐tubulin‐N‐acetyltransferase 1 (ATAT1/MEC‐17), and the Tip60/Fe65 complex—have been implicated in its acetylation [32, 33, 34, 35]. Non‐enzymatic acetylation in the presence of acetyl‐CoA has also been reported [36]. Mass spectrometry has identified over 20 acetylation sites in the tandem central repeat region in both human and murine cortactin [37, 38, 39, 40, 41, 42, 43], with more than 60% of lysines in this region being modifiable [34]. The acetylation status of this segment is a key determinant of F‐actin binding: acetylation‐mimicking (K→Q) mutations impair binding, while deacetylation‐mimicking (K→R) mutations enhance it [34].

On the KDAC side, HDAC6 and SIRT1 have been identified as CTTN deacetylases and/or interaction partners in several independent cell‐based studies using their genetic manipulation and/or pharmacologic inhibition [34, 41, 44, 45, 46, 47, 48, 49, 50, 51, 52, 53, 54, 55]. Both DD1 and DD2 catalytic domains of HDAC6 have been reported to deacetylate the repeat region of CTTN thus modulating actin binding and cell motility [34]. Similarly, SIRT1 has been found to deacetylate cortactin in a NAD^+^‐dependent manner, potentially influencing tumor invasion and metastasis [35]. Other studies implicated HDAC8 and SIRT2 in regulating cortactin acetylation levels; however, the precise mechanisms and targeted residues remain mostly unclear [34, 39, 43, 46, 56, 57].

While cell‐based studies have provided invaluable insights into how various KDACs regulate CTTN acetylation and the resulting biological effects, they leave several key questions unanswered. For instance, it remains unclear whether different KDACs act on the same lysine residues or display distinct substrate specificities. Moreover, it is not known whether enzymes such as HDAC8 and SIRT2 deacetylate CTTN directly, or if their effects on CTTN acetylation are mediated indirectly through the regulation of other components of the (de)acetylation machinery. To directly address these questions, we used genetic code expansion (GCE) to generate seven site‐specifically acetylated cortactin variants (K87, K124, K161, K198, K235, K272, and K309), representing conserved and frequently acetylated sites within the central tandem repeat region, and assessed their deacetylation by various KDACs using purified components *in vitro*. To our knowledge, this is the first comprehensive analysis of site‐specific CTTN deacetylation by multiple human KDACs, providing mechanistic insights into their substrate preferences and laying the groundwork for understanding how these enzymes fine‐tune cytoskeletal dynamics via cortactin regulation.

## Results

### Production and characterization of wild‐type CTTN and its acetylated variants

**Fig. 1:**
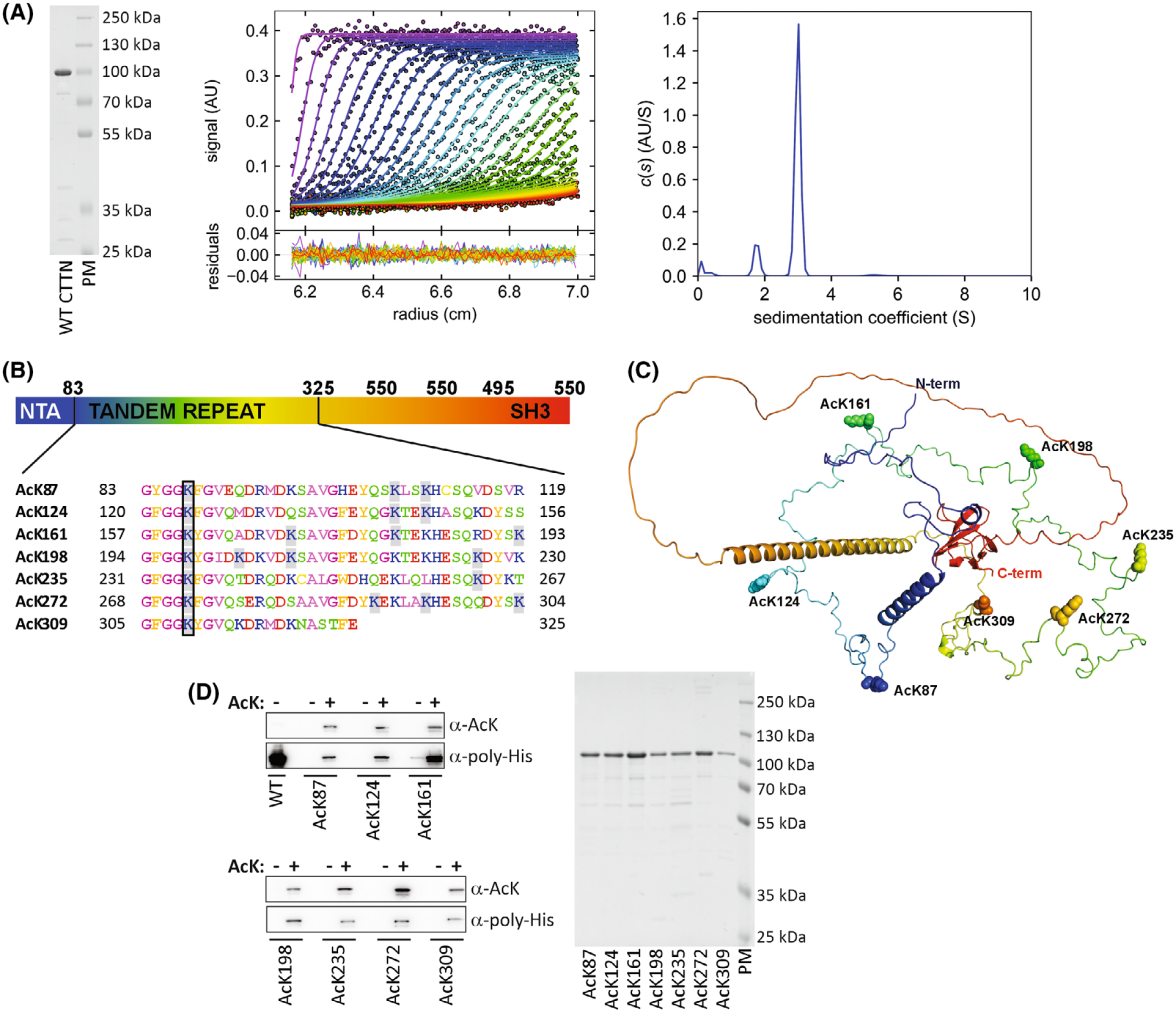
CTTN domain structure, purification, and characterization. (A): Purification and AUC characterization of wild‐type full‐length CTTN. On the left side, a representative CBB‐stained SDS/PAGE gel shows purified wild‐type CTTN (PM – molecular weight marker). The chart in the center reveals fitted sedimentation velocity data, while the right panel contains the c(s) distribution of the CTTN sample. (B): Schematic representation of CTTN (human isoform 1; Uniprot accession Q14247‐1) with the tandem repeat sequences harboring acetylation sites (sites studied here in black box). Lysine residues highlighted in gray represent the known *in vivo* acetylation sites of cortactin ([37, 39, 41, 42] and curated MS data deposited in the PhosphoSitePlus database [38]); NTA – N‐terminal acidic domain; SH3 – Src homology 3 domain. The alignment was performed manually using the published tandem repeat sequences. (C): AlphaFold model of human CTTN (cartoon representation; AF‐Q14247‐F1) with AcK sites shown as balls. The tandem repeats are predicted to be situated in the intrinsically disordered regions of the protein. The model is colored in a rainbow blue‐red gradient (from N to C terminus) created with pymol software. (D): Representative western blot analysis of cell lysates demonstrates successful incorporation of N^ɛ^‐acetyl‐L‐lysine (AcK) at defined positions in response to amber (UAG) stop codons, and the production of full‐length AcK‐CTTN only in the presence of externally supplied AcK (left). Representative SDS/PAGE of AcK‐CTTN variants purified via a tandem affinity chromatography (right).

The gene encoding wild‐type full‐length human CTTN was flanked by the Twin‐Strep‐tag and His_8_‐tag at 5′ and 3′ ends, respectively, and cloned into the pCDF‐PylT plasmid (Fig. S1). The recombinant fusion protein was then expressed in *E. coli* and purified to homogeneity using a tandem two‐step affinity chromatography on Ni‐NTA and Strep‐Tactin columns, yielding approximately 250 μg per liter of culture. The protein preparation was analyzed by analytical ultracentrifugation to confirm monodispersity and correct foldedness. A sedimentation velocity experiment revealed a predominant peak corresponding to the CTTN monomer together with the absence of higher molecular mass aggregates. The determined sedimentation coefficient of 3.2 S (s_20,w_) is in good agreement with the previously reported value and suggests a similar protein shape/folding [58] (Fig. 1A).

Site‐directed mutagenesis was used to insert the amber TAG stop codons at positions K87, K124, K161, K198, K235, K272, and K309 of the wild‐type CTTN (Fig. 1B,C). CTTN variants harboring a single N^ε^‐acetyl‐L‐lysine (AcK) residue at a defined position were expressed in *E. coli* by the genetic expansion technology using an orthogonal pair of acetyl‐lysyl‐tRNA synthetase 3 (AcKRS3) [59] and tRNA_CUA_^PylU25C^ [60]. CTTN expression and incorporation of AcK into permissive sites were initially analyzed by western blotting in cell lysates using α‐AcK and α‐polyHis antibodies. Robust acetylation‐specific signals were observed only upon supplementation of the growth medium with 10 mM AcK, confirming the presence of AcK residues as well as successful expression of acetylated proteins (AcK‐CTTN; Fig. 1D, left panel). The optimized tandem affinity purification protocol typically yielded between 5 and 20 μg of AcK‐CTTN per liter of *E. coli* culture—a 13‐ to 50‐fold reduction compared to the wild‐type CTTN—but still mostly sufficient for downstream biochemical experiments (Fig. 1D). Purified proteins were further analyzed by mass spectrometry and the MS/MS data of tryptic digests confirmed the presence of the acetylated lysine residue at expected positions in all AcK‐CTTN variants (Fig. S2).

### Screening of AcK‐CTTN deacetylation by HDACs 1–11

**Fig. 2:**
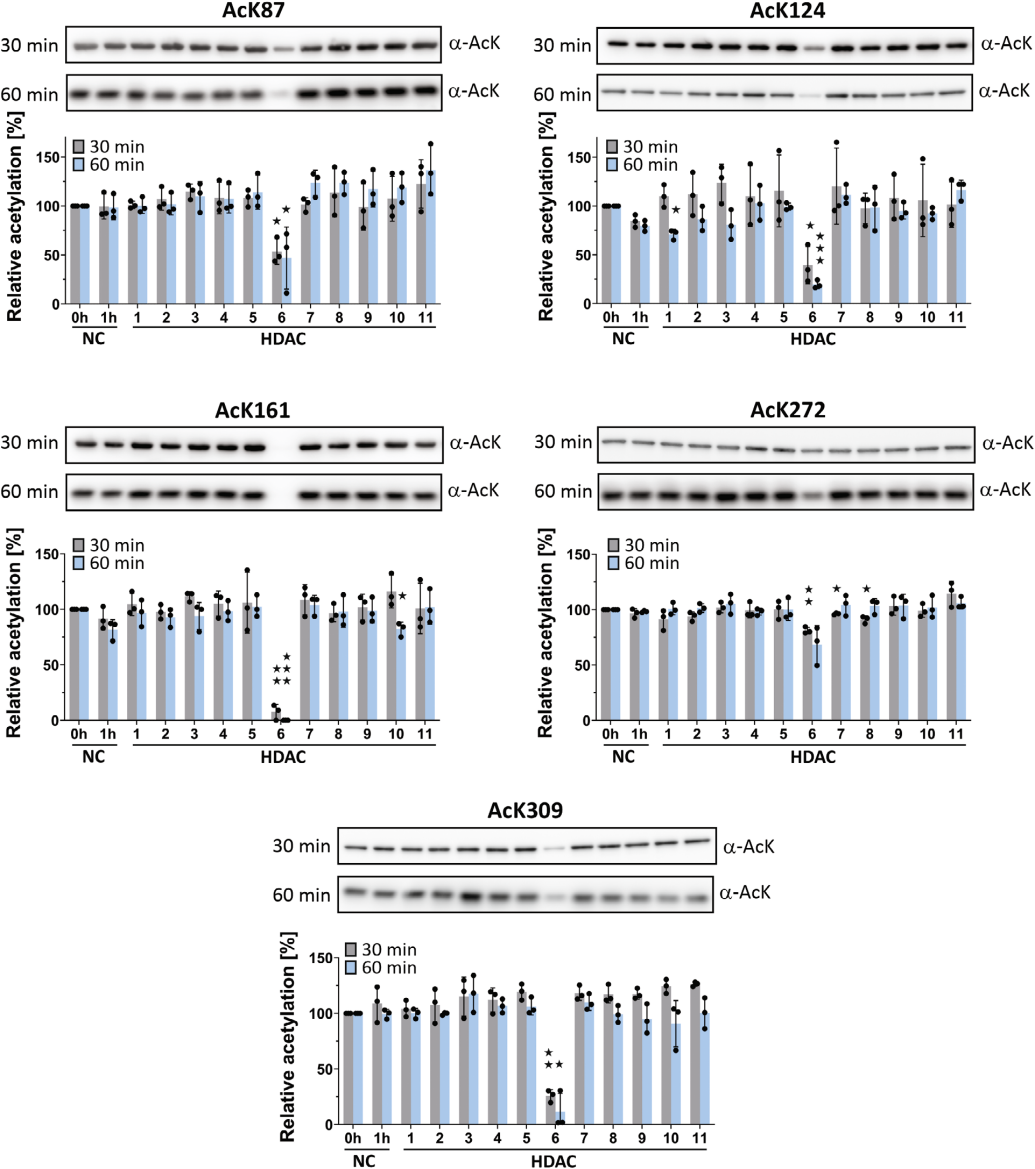
Deacetylation of AcK‐CTTN variants by human HDACs 1–11. AcK‐CTTNs (0.5 μm) were incubated with purified HDACs (50 nm) at 37 °C. Aliquots were taken at 30 and 60 min, separated by SDS/PAGE, and visualized by immunoblotting (IB) using α‐AcK antibody and signals quantified with Quantity One 1‐D Analysis software. Bar graphs represent mean ± SD. from three independent experiments. Statistical significance was determined using a paired t‐test (*p < 0.05, **p < 0.01, ***p < 0.001). Negative controls (NC, no enzyme) at 0 and 60 min confirmed no spontaneous deacetylation. Of all HDACs tested, only HDAC6 induced a marked decrease in AcK‐CTTN acetylation signal. Representative immunoblots and full replicate data are provided in the Supporting Information (Fig. S3).

For initial screening, five AcK‐CTTN variants with the highest expression levels (AcK87, AcK124, AcK161, AcK272, and AcK309) were tested against a panel of enzymatically active purified human HDACs 1–11 [61, 62, 63, 64]. Acetylation assays were conducted at a 10 : 1 substrate‐to‐enzyme molar ratio at 37 °C for 30 and 60 min, and reactions were monitored by immunoblotting using the α‐AcK antibody. All HDACs were tested individually, except for HDAC3, which was also included as part of an active binary complex with NCOR2 [65, 66]. As shown in Figs 2 and S3, HDAC6 was the only isoform to consistently and significantly reduce AcK‐CTTN acetylation levels, confirming its known role as a cortactin deacetylase in a fully reconstituted, purified *in vitro* system. A reduction or complete loss of α‐AcK signal was observed across all tested variants, suggesting that HDAC6 efficiently recognizes and deacetylates lysines located at conserved positions within the cortactin tandem repeats (residues 87 + 37 n, where *n* = 0–6).

Interestingly, despite marked sequence similarities, individual variants exhibited differing rates of deacetylation. AcK161 was fully deacetylated within 30 min, whereas other sites retained partial acetylation even after 60 min. Surprisingly, none of the other zinc‐dependent HDACs, including HDAC8 previously suggested to act on cortactin [39, 43, 56, 57], showed any detectable deacetylation activity under our assay conditions. These findings highlight HDAC6 as the predominant cortactin deacetylase among the class I, II, and IV HDACs tested *in vitro*.

### AcK‐CTTN deacetylation by HDAC6, HDAC8, SIRT1, and SIRT2

**Fig. 3:**
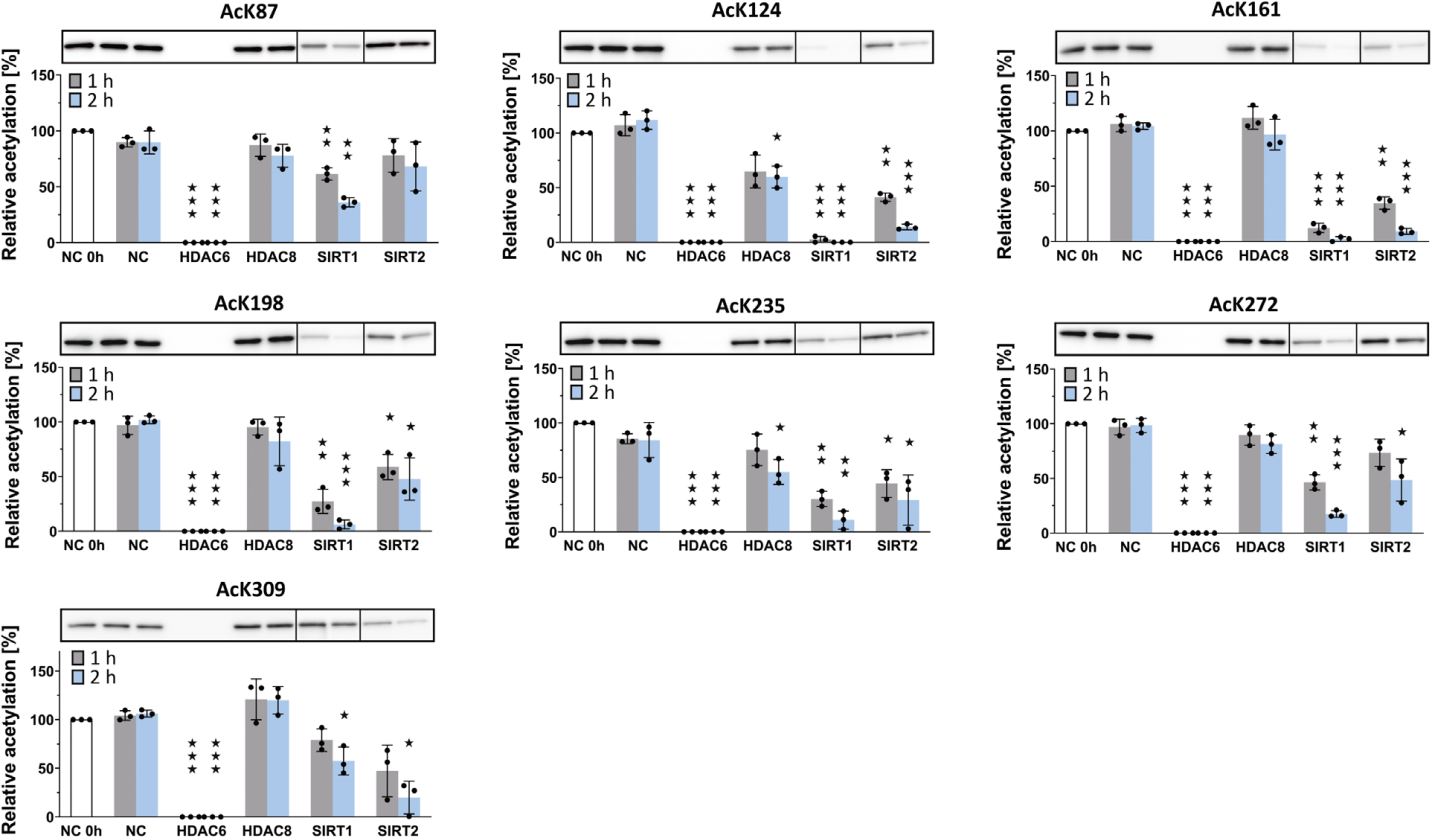
Cortactin deacetylation by HDAC6, HDAC8, SIRT1, and SIRT2. Western blot analysis of AcK‐CTTNs incubated with HDAC6, HDAC8, SIRT1, or SIRT2 at 37 °C for 60 and 120 min. Reaction aliquots were separated by SDS/PAGE, electrotransferred to a PVDF membrane, and probed with the α‐AcK antibody with chemiluminescence detection. Signals quantified with Quantity One 1‐D Analysis software and bar graphs represent mean ± SD from three independent experiments. Statistical significance was determined using a paired *t*‐test (*p < 0.05, **p < 0.01, ***p < 0.001). Representative immunoblots and full replicate data are provided in the Supporting Information (Fig. S4).

We next focused in more detail on HDAC6, HDAC8, SIRT1, and SIRT2, the four KDACs previously implicated in cortactin deacetylation in cell‐based studies [34, 35, 39, 41, 44, 45, 46, 47, 48, 49, 50, 51, 52, 53, 54, 55, 56, 57, 67]. To improve detection sensitivity and account for the lower catalytic activity of HDAC8, SIRT1, and SIRT2, we extended the reaction time to 2 h and increased enzyme concentrations to equimolar substrate‐to‐enzyme ratios. The incubation of all seven AcK‐CTTN variants with HDAC6 led to complete deacetylation at each tested site, including AcK198 and AcK235, which were omitted from the initial screen due to low expression yields (Figs. 3 and S4). In contrast, under high enzyme concentration and long incubation time conditions, very weak deacetylation activity of specific AcK‐CTTN variants was detected for HDAC8, another zinc‐dependent HDAC.

**Fig. 4:**
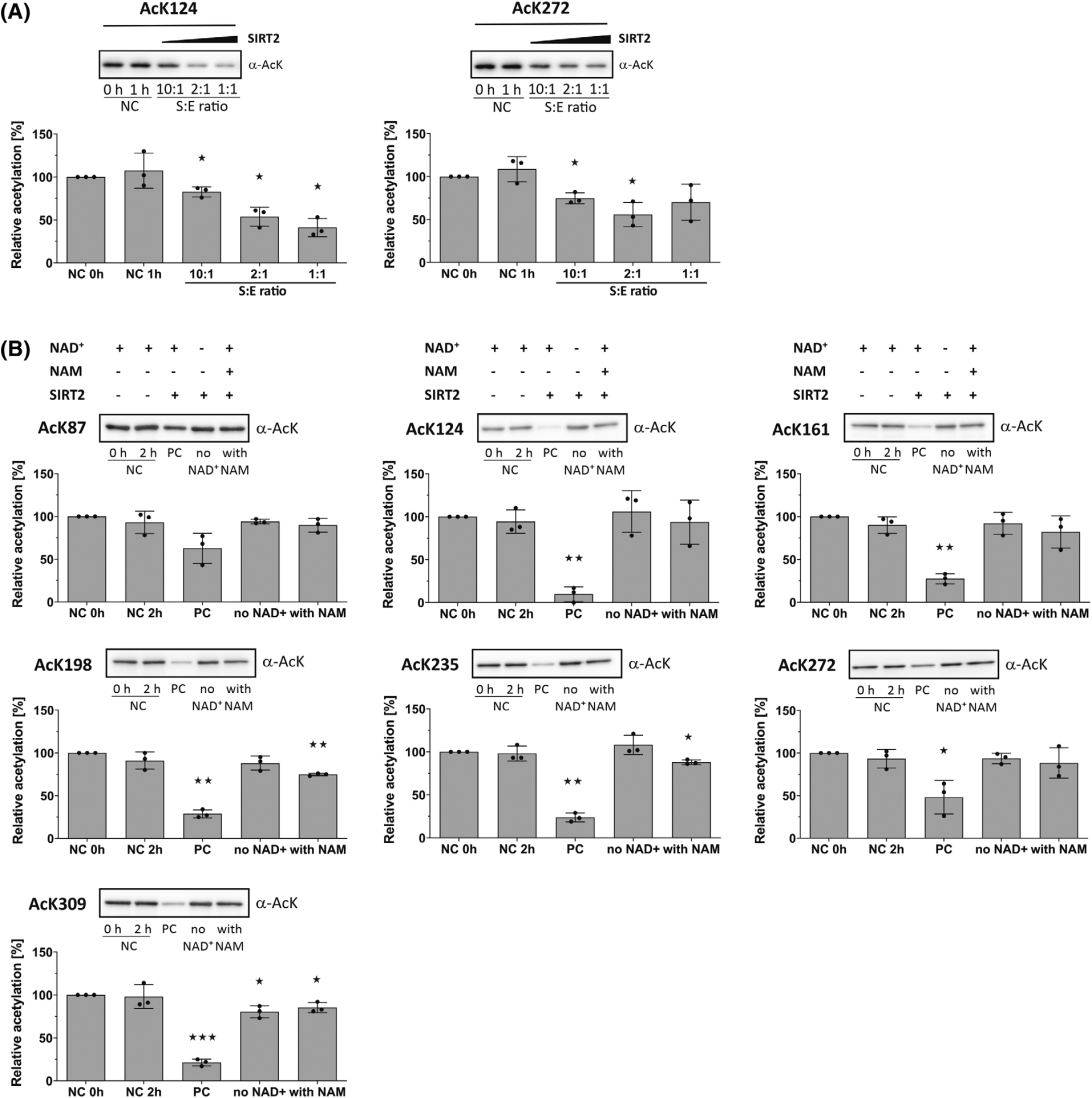
Cortactin deacetylation by human SIRT2. AcK‐CTTNs variants were incubated with human SIRT2 at 37 °C for 60 (A) and 120 min (B). Reaction aliquots were separated by SDS/PAGE, electrotransferred to a PVDF membrane, and probed with the α‐AcK antibody with chemiluminescence detection. Signals quantified with Quantity One 1‐D Analysis software and bar graphs represent mean ± SD from three independent experiments. Statistical significance was determined using a paired t‐test (*p < 0.05, **p < 0.01, ***p < 0.001).(A) Concentration‐dependent deacetylation of AcK124‐CTTN and AcK272‐CTTN by SIRT2. WB analysis was performed as described above with SIRT2 concentrations, 0.04, 0.2, and 0.4 μm. (B) The effect of NAD^+^ and NAM (10 mm concentrations) on SIRT2‐catalyzed deacetylation of AcK‐CTTN variants. Representative immunoblots and full replicate data are provided in the Supporting Information (Fig. S5).

As for sirtuins, recombinant SIRT1 was clearly active and deacetylated all tested AcK‐CTTN variants, with a preference for AcK124, AcK161, AcK198, and AcK235. Under identical conditions, SIRT1 was consistently more efficient than SIRT2, yet its activity remained substantially lower than that of HDAC6. SIRT2 also displayed time‐ and concentration‐dependent deacetylation activity toward all seven variants, but with lower efficacy than SIRT1 and HDAC6 (Figs 3, 4 and Figs S4, S5). This activity was strictly NAD^+^‐dependent and was abolished in the presence of 10 mm nicotinamide (a sirtuin inhibitor), confirming both the enzymatic nature and specificity of the reaction (Fig. 4). These results provide the first direct biochemical evidence for SIRT2 as a bona fide cortactin deacetylase.

### Deacetylation of substrate‐derived peptides

**Fig. 5:**
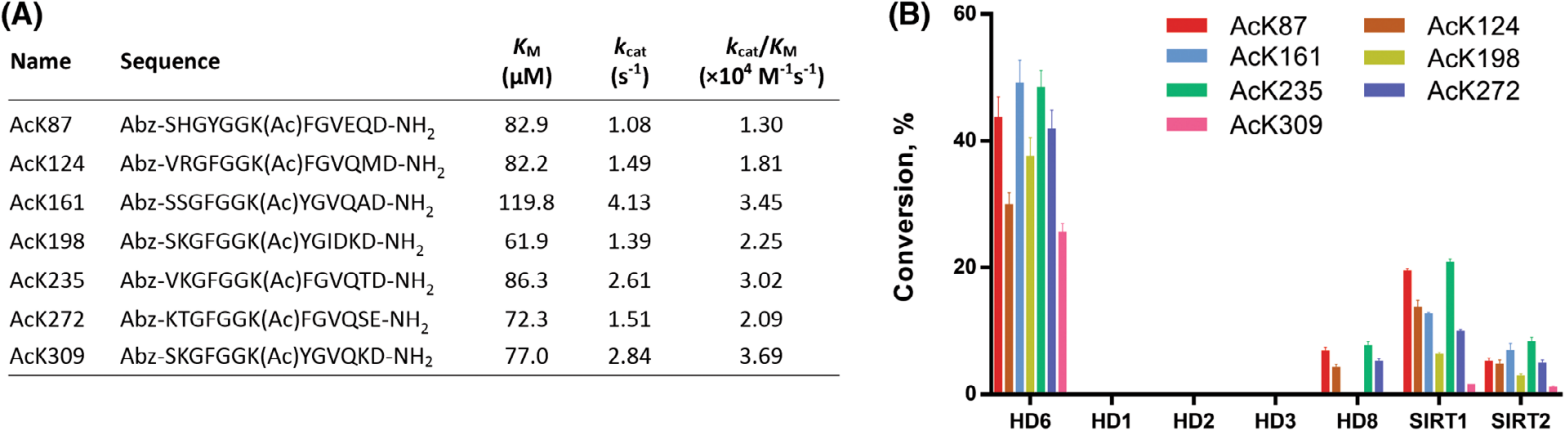
Deacetylation of AcK‐CTTN‐derived peptides by selected HDACs. (A) Peptide sequences and corresponding kinetic parameters derived from the nonlinear regression fit of experimental data shown in Supplementary Table S1. Peptides (two‐fold dilution series) were incubated with full‐length HDAC6 at 37 °C for 30 min and the deacetylation levels determined by HPLC. (B) Deacetylation activity of selected HDACs on AcK‐CTTN‐derived peptides. 20 μm peptides were incubated with selected HDACs at 37 °C for 30 min and the deacetylation levels determined by an HPLC‐based assay. HDAC6 was the most efficient deacetylase, followed by SIRT1 and SIRT2. HDAC8 exhibited low but reproducible deacetylation activity toward a subset of peptides. HDAC1, 2, and 3 showed no detectable activity on these peptides. Bar graphs represent the mean ± SD from three independent experiments.

To compare KDAC substrate preferences at the protein and peptide levels, we synthesized 13‐mer acetylated peptides corresponding to the sequences flanking the seven acetylated lysines studied in cortactin (Fig. 5A) and assessed their deacetylation by selected HDACs using RP‐HPLC. HDACs 4, 5, 7, 9, 10, and 11 were excluded due to either reported inactivity toward acetylated peptides (HDACs 4, 5, 7, 9, 11) or preference for small‐molecule substrates (HDAC10) [68]. Peptides were incubated with defined enzyme concentrations identical for each HDAC/peptide pair (Table S1) at 37 °C for 30 min, and reaction products were quantified by fluorescence‐coupled HPLC. Consistent with the data on full‐length AcK‐CTTN, HDAC1, 2, and 3 showed no detectable activity on these peptides. HDAC6 was again the most efficient deacetylase, followed by SIRT1 and SIRT2. For HDAC6, the apparent K_M_ values ranged from 62 to 120 μm (lowest for AcK198 and highest for AcK161), suggesting modest affinity for these isolated peptide sequences. Among them, AcK161 was deacetylated most efficiently, with a k_cat_ of 4.12 s^−1^—consistent with its rapid turnover as a full‐length substrate (Figs 5 and S6). Notably, HDAC8, which showed very weak activity at AcK124 and AcK235 of full‐length AcK‐CTTN (compare Fig. 3), exhibited low but reproducible deacetylation activity toward a subset of peptides, reaching approximately 10–15% of HDAC6 activity and activity comparable to SIRT2 (Fig. 5B). These results indicate that HDAC8 can act directly on some cortactin sequences in a peptide context, although its activity may be influenced by structural elements present in the full‐length AcK‐CTTNs.

Importantly, the peptide‐based experiments should be interpreted with caution, as enzyme activity in cells is influenced by additional factors such as complex formation and the spatial and temporal distribution of enzyme–substrate pairs [69]. Because HDACs do not exhibit strict substrate specificity at the peptide level, high enzyme concentrations *in vitro* can drive deacetylation of many peptide sequences, albeit with differing efficiencies. This is illustrated in Fig. S7, where we compared deacetylation of AcK‐CTTN peptides with H3K9‐ and p53K382‐derived peptides. At high enzyme concentrations, HDAC1–3 preferentially deacetylated their canonical substrates but were also able to deacetylate AcK‐CTTN peptides, though less efficiently. By contrast, HDAC8 showed a preference for AcK‐CTTN peptides under the same conditions, and SIRT1 and SIRT2 were similarly active against CTTN‐ vs p53 and H3K9‐derived peptides. Together, these data suggest that cortactin‐derived peptides can in some cases serve as useful surrogates for studying HDAC6 substrate preferences. More generally, however, KDAC substrate recognition is likely to depend on the structural context, and results obtained with short peptides should not be overinterpreted, as also highlighted in previous work [11, 70].

### Deacetylation of AcK‐CTTN and AcK‐CTTN‐derived peptides by HDAC6 variants

**Fig. 6:**
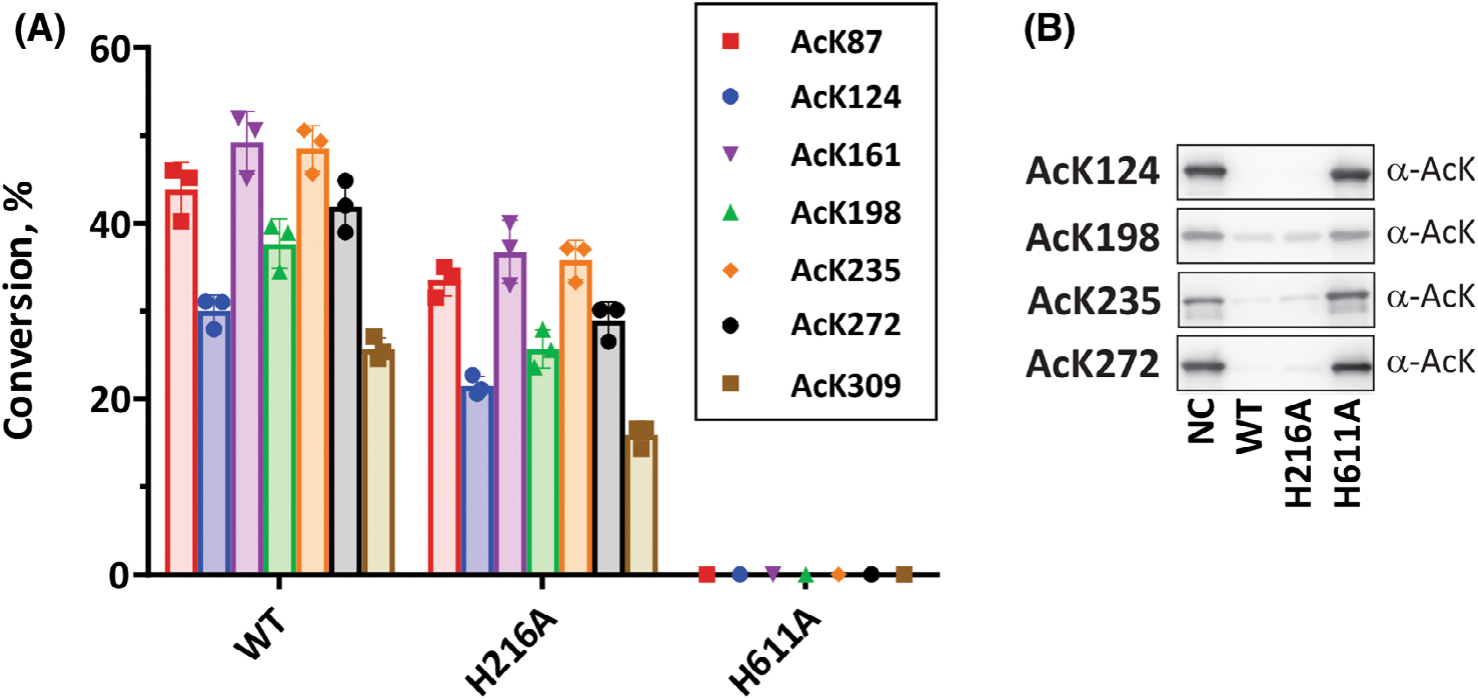
Deacetylation of AcK‐CTTN and AcK‐CTTN‐derived peptides by wild‐type and mutant HDAC6 variants. (A) Deacetylation of cortactin‐derived peptides by HDAC6 variants. Peptides (20 μm) were incubated with wild‐type HDAC6 or mutants H216A (inactive DD1) and H611A (inactive DD2) at 37 °C for 30 min. Deacetylation levels were determined by RP‐HPLC. Wild‐type and H216A HDAC6 showed comparable activity, while the H611A mutant was inactive. Bar graphs represent the mean ± SD from three independent experiments. (B) Deacetylation of selected full‐length AcK‐CTTN variants. AcK‐CTTN proteins (0.5 μm) were incubated with 250 nM HDAC6 variants at 37 °C for 2 h. Acetylation levels were assessed by immunoblotting using the α‐AcK antibody; Ponceau S staining served as a loading control (Fig. S8). All tested CTTN variants (AcK124, AcK198, AcK235, AcK272) were deacetylated by wild‐type HDAC6 and the H216A mutant, while the H611A mutant showed no activity. NC ‐ negative control (no HDAC). The blot shown is representative of *n* = 3 independent experiments.

To determine which catalytic domain of HDAC6 is responsible for cortactin deacetylation, we compared the activity of full‐length wild‐type HDAC6 with its catalytically inactivated variants: H216A (inactive DD1) and H611A (inactive DD2). When tested against both full‐length AcK‐CTTN variants and their corresponding acetylated peptides, the H216A mutant retained deacetylation activity across all substrates, indicating that the DD2 domain is catalytically competent and primarily responsible for cortactin deacetylation. In contrast, the H611A mutant exhibited no activity, confirming that the DD1 domain alone cannot catalyze deacetylation of cortactin substrates *in vitro*. These results were consistent across both substrate types (Figs 6 and S8), reinforcing that HDAC6's DD2 domain plays the dominant role in recognizing and deacetylating lysines within the cortactin repeat region.

## Discussion

In this study, we investigated the role of human lysine deacetylases (KDACs) in regulating the acetylation of cortactin, a key actin‐binding protein involved in cytoskeletal remodeling. While previous cell‐based studies have linked specific KDACs to cortactin deacetylation, they often relied on genetic or pharmacological manipulation, leaving open questions about direct enzymatic activity and site specificity. To address this, we employed a fully reconstituted *in vitro* system using purified components and site‐specifically acetylated cortactin variants. Our goal was to provide direct and unbiased evidence identifying which KDACs can deacetylate cortactin and to map their activity to individual lysine residues within the central tandem repeat region.

Using genetic code expansion (GCE), we successfully expressed and purified seven AcK‐CTTN variants, each acetylated at a defined lysine within the tandem repeat region. These sites represent some of the most frequently reported cortactin acetylation positions and are conserved across repeat units [34, 37, 39, 40, 41, 42]. As commonly observed with the incorporation of unnatural amino acids (UAAs), expression yields of AcK‐CTTN variants were significantly lower (approximately 13–50‐fold) compared to wild‐type cortactin, likely due to premature translation termination. Interestingly, we also detected substantial expression of N‐terminally truncated cortactin “by‐products,” which appeared only after IPTG induction and also in the absence of external AcK. These data suggest that the truncated variants are most likely the CTTN degradation products with the added possibility that some of them are as well products of translation reinitiation following ribosomal stalling at the amber codon (Fig. S9). In fact, the latter phenomenon of translation reinitiation has been, in the context of GCE, previously reported in bacterial, yeast, and mammalian systems [71, 72, 73, 74]. These findings underscore the importance of the construct design and purification strategies; in particular, the use of distinct N‐ and C‐terminal affinity tags is critical for distinguishing full‐length protein from truncated by‐products.

Our *in vitro* findings confirm that HDAC6 directly deacetylates cortactin, in agreement with numerous cell‐based studies [34, 41, 44, 45, 46, 47, 50, 51, 52, 53, 67]. Importantly, we show for the first time that HDAC6 targets all tested acetylation sites within the cortactin tandem repeat region, albeit with somewhat varied efficiency. It is worth noting that there is a high degree of sequence conservation in the CTTN repeats (54–81% identity) and the sequences closely match the optimal HDAC6 recognition sequence, with conserved glycine residues at −1 and −2 positions and bulky aromatic residues (phenylalanine or tyrosine) at the +1 position [63]. Using inactivating mutations in HDAC6's catalytic domains, we further demonstrated that the DD2 domain is solely responsible for cortactin deacetylation *in vitro*. These results differ from previous cellular studies reporting that inactivation of either DD1 or DD2 reduces cortactin acetylation [45, 50, 52]. One possible explanation is that the H216A mutation used to inactivate DD1 may affect the overall stability and thus the resulting specific activity of HDAC6 rather than be directly involved in substrate deacetylation. More broadly, our data contribute to the ongoing debate over the functionality of HDAC6's dual catalytic domains [16, 75, 76, 77, 78]. Prior studies using peptide libraries suggest that DD2 accommodates a wide range of internal acetyl‐lysine substrates, whereas DD1 has narrower specificity, favoring substrates with C‐terminal acetylation [62, 79]. Interestingly, Saito *et al*. proposed that DD1 may also act on acetylated lysines located within intrinsically disordered regions (IDRs) [52]. Notably, all cortactin acetylation sites examined here also reside within predicted IDRs (Fig. 1C), yet none were targeted by DD1 in our assays. This suggests that DD1 activity toward IDR‐resident lysines is not a general rule and may depend on additional contextual or structural factors. Further biochemical studies will be needed to resolve these domain‐specific substrate preferences for individual HDAC6 substrates.

In addition to HDAC6, our study provides the first direct biochemical evidence that SIRT2 can deacetylate cortactin *in vitro*. This activity was time‐, concentration‐, and NAD^+^‐dependent, consistent with SIRT2's known enzymatic mechanism. Previous evidence for SIRT2 involvement in cortactin regulation was based solely on cell‐based assays, such as SIRT2 knockdown studies in A549 lung epithelial cells and neurons [34, 46]. On the other hand, Zhang *et al*. failed to detect a direct interaction between SIRT2 and cortactin, leading to the hypothesis that SIRT2 might act indirectly through other deacetylases [35]. Our results clarify this uncertainty and underscore the importance of using complementary biochemical and cellular approaches to confidently assign enzyme–substrate relationships [80]. Interestingly, HDAC6 and SIRT2 both target overlapping sites in the cortactin repeat region and have been shown to play a common (and potentially functionally redundant) role in regulating similar biological processes, such as neuronal migration and cancer cell invasion [46, 81]. The reported physical interaction between these two enzymes [82, 83] further supports potential functional cooperation. While SIRT2 was less efficient than HDAC6 and SIRT1 in our *in vitro* assays, this does not rule out its biological relevance, as cellular context and interacting partners may enhance SIRT2 activity and/or specificity.

In addition to SIRT2, our data demonstrate that SIRT1 also directly deacetylates cortactin at multiple sites. Across all AcK‐CTTN variants tested, SIRT1 displayed higher catalytic efficiency than SIRT2, although it remained substantially less active than HDAC6. The activity profile of SIRT1, intermediate between HDAC6 and SIRT2, suggests that the enzyme likely contributes to cortactin regulation under specific cellular conditions in which SIRT1 is locally enriched or functionally recruited [35, 48, 49, 54, 55, 84]. These observations indicate that both sirtuins participate in fine‐tuning cortactin acetylation, albeit to different extents. Finally, our *in vitro* data do not support a major direct role for HDAC8 in cortactin deacetylation, despite several cell‐based studies suggesting such involvement [39, 56, 57]. This discrepancy underscores the well‐recognized limitations of comparing purified biochemical assays with cellular acetylation profiles. For example, Knyphausen *et al*. showed that nearly half of putative cellular sirtuin substrates fail to undergo deacetylation *in vitro*, highlighting the strong influence of protein context, posttranslational modifications, and cofactor availability on substrate recognition [7]. Conversely, our *in vitro* data reveal AcK161 as one of the most efficiently turned‐over sites by HDAC6, whereas Ito *et al*. reported the strongest accumulation of acetylation at K309 in cells treated with broad‐spectrum KDAC inhibitors [85]. These differences likely reflect the impact of cortactin's conformational dynamics, phosphorylation state, and interactions with binding partners, which together modulate lysine accessibility and the recruitment of specific KDACs [86, 87]. Additionally, pan‐acetyllysine antibodies may recognize AcK residues not directly targeted in biochemical reconstitutions, such as AcK144‐CTTN in the case of cortactin [43] Thus, although biochemical reconstitution is essential for defining direct enzyme–substrate relationships and intrinsic site selectivity, *in vivo* acetylation patterns arise from a broader regulatory network that cannot be fully captured by *in vitro* assays.

## Conclusions

Despite two decades of research, the structural dynamics and regulatory mechanisms governing cortactin function, particularly the roles of its posttranslational modifications, remain incompletely understood. Using genetically encoded, site‐specifically acetylated cortactin variants, we define the intrinsic activities of major human KDACs toward the cortactin repeat region. HDAC6 is the most likely the predominant deacetylase, acting through its DD2 domain, with SIRT1 and SIRT2 contributing weaker but measurable activities. Other KDACs showed little or no activity, indicating limited direct involvement. These results provide a biochemical foundation for understanding cortactin deacetylation and offer a framework for interpreting its regulation in cellular contexts.

## Materials and methods

### Cloning and site‐directed mutagenesis

pAcKRS3 was provided by Dr. Jason Chin (MRC Laboratory of Molecular Biology, Cambridge, UK), the pCDF‐PylT plasmid was provided by Dr. Eyal Arbely (Ben Gurion University, Beersheva, Israel) [59, 88].

The sequence encoding human CTTN (isoform 1; Uniprot Q14247‐1) was codon optimized for expression in *E. coli* and commercially synthesized by GenScript. The synthetic cortactin gene, flanked by Twin‐Strep‐tag (5′ end) and His‐tag coding sequence (3′ end), was cloned into the pCDF_PylT plasmid (Fig. S1). Plasmids for expression of AcK‐CTTN variants (positions K87, K124, K161, K198, K235, K272, and K309) were generated by site‐directed mutagenesis using a QuikChange protocol (Agilent Technologies) and pairs of mutagenic primers listed in Table S2. Introduction of in‐frame TAG mutations at permissive positions in the cortactin gene was verified by Sanger sequencing.

### Expression and purification of cortactin variants

Non‐acetylated CTTN was expressed in *E. coli* Rosetta(DE3) cells harboring the pCDF‐PylT‐*cttn* plasmid. Cells were cultured in LB medium supplemented with 100 μg·ml^−1^ spectinomycin at 37 °C. At OD_600_ of ~ 0.5, protein expression was induced by the addition of 0.5 mM IPTG and continued at 30 °C for 4 h. The acetylated cortactin variants were expressed in Rosetta(DE3) cells co‐transfected by pAcKRS3 and pCDF‐PylT‐*cttn*_KxTAG (*x* = 87, 124, 161, 198, 235, 272 or 309) plasmids. The cells were grown in LB medium containing 50 μg·ml^−1^ kanamycin and 100 μg·ml^−1^ spectinomycin at 37 °C. At OD_600_ of ~ 0.3–0.4, the growth medium was supplemented with 10 mm N^ε^‐acetyl‐L‐lysine (GL Biochem, Shanghai, China) and 20 mm nicotinamide (Sigma‐Aldrich, Saint Louis, MO, USA). The expression was induced 30 min later and performed as described above.

Four hours post induction, cells were harvested by centrifugation (15 min/6000 ***g***/4 °C) and pellets were resuspended in 20 mm Tris, 150 mm NaCl, pH 8, supplemented with the complete EDTA‐free cocktail of protease inhibitors (Roche, Basel, Switzerland). Cells were lysed by sonication (Q700 sonicator, Qsonica, Newtown, CT, USA); CHAPS was added to a final concentration of 0.1% (w/v), and samples were centrifuged (30 min/30 000 ***g***/4 °C) and filtered (0.45 μm). CTTN/AcK‐CTTN variants were purified by the combination of Ni‐NTA and Strep‐Tactin affinity chromatography using gravity‐flow columns. Briefly, a cleared lysate was loaded onto a Ni‐NTA Superflow resin (IBA) equilibrated with 20 mm Tris–HCl, 150 mm NaCl, 50 mm imidazole, 0.1% CHAPS, pH 8. After extensive washing with the equilibration buffer, bound His‐tagged proteins were eluted with 20 mm Tris–HCl, 150 mm NaCl, 0.5 m imidazole, 0.1% CHAPS, pH 8. The elution fractions were diluted with 20 mm Tris–HCl, 150 mm NaCl, 0.1% CHAPS, pH 8 to decrease imidazole concentration to 150 mm, and the sample was immediately applied onto Strep‐Tactin Superflow resin (IBA). After thorough washing with 20 mm Tris–HCl, 150 mm NaCl, 0.1% CHAPS, pH 8, CTTN/AcK‐CTTN proteins were eluted with 20 mm Tris–HCl, 150 mm NaCl, 0.1% CHAPS, 10 mm desthiobiotin, pH 8. Sample purity was verified by SDS/PAGE and proteins concentrated using an Amicon Ultra centrifugal concentrator with a 3 kDa cut‐off limit (Merck Millipore, Darmstadt, Germany). Purified proteins were flash‐frozen in liquid nitrogen and stored at −80 °C. CTTN/AcK‐CTTN concentration was determined spectrophotometrically at 280 nm (Nanodrop One, Thermo Scientific, Waltham, MA, USA). Alternatively, for low concentrated and/or partially‐purified samples, we employed a quantitative densitometric analysis of Coomassie brilliant blue G‐250‐stained SDS/PAGE gel in quantityone software (Bio‐Rad, Hercules, CA, USA). For this quantification, purified CTTN of known concentration (determined by spectrophotometry) was used to generate a calibration curve.

### Analytical ultracentrifugation

Analytical ultracentrifugation was carried out on a ProteomeLab XL‐I analytical ultracentrifuge (Beckman Coulter, Brea, CA, USA) equipped with An‐60 Ti rotor. Prior to analysis, non‐acetylated cortactin was dialyzed against 20 mm Hepes, 140 mm NaCl, 10 mm KCl, 1 mm TCEP, pH 7.4, and the corresponding dialysate served as a reference solution. A sedimentation velocity experiment was conducted at 20 °C using a titanium double‐sector centerpiece cell with a 12 mm optical pathlength (Nanolytics Instruments, Potsdam, Germany). Cell sectors were filled with 380 μl of non‐acetylated CTTN (4.8 μm) and a reference buffer, respectively. Following a 2 h temperature equilibration, the sample was centrifuged at 48 000 rpm, and the sedimentation was monitored by absorbance optics at 280 nm. Data were acquired in continuous scan mode with a spatial resolution of 0.003 cm and scan intervals of 5 min. The partial specific volume of cortactin, and the buffer density and viscosity were estimated from the amino acid sequence and buffer composition using Sednterp 3 [89]. The sedimentation profiles were analyzed using Sedfit 18.1 [90] with the c(s) distribution model and maximum entropy regularization on a confidence level of 0.68. The figures were created in GUSSI 2.4.6 [91].

### Expression and purification of zinc‐dependent HDACs

Expression of human HDAC1–11 and mutant HDAC6 constructs was carried out as previously described [62, 64, 92]. Briefly, suspension culture of HEK‐293 T cells (kindly provided by Dr. Ondrej Vanek, Faculty of Science, Charles University, Prague, Czech Republic; RRID: CVCL_0063) was transiently transfected with HDAC expression plasmids using linear polyethyleneimine (PEI). Cells were harvested 72 h post‐transfection by centrifugation (500 ***g***, 10 min, 4 °C), and the resulting pellets were resuspended in lysis buffer (50 mm Tris–HCl, 10 mm NaCl, 5 mm KCl, 2 mm MgCl_2_, 10% glycerol, pH 8) supplemented with 2 U·ml^−1^ benzonase (Merck) and a protease inhibitor cocktail (Roche). Cells were lysed by sonication (2 × 10 s), followed by the addition of Igepal‐630 to a final concentration of 0.2%. The lysate was incubated on ice for 30 min, then adjusted to 150 mm NaCl (using 4 m NaCl stock solution) and clarified by centrifugation (40 000 g, 30 min, 4 °C). All experiments were performed with Mycoplasma‐free cells.

The supernatant was loaded onto a Strep‐Tactin column (IBA) pre‐equilibrated with 50 mm Tris–HCl, 150 mm NaCl, 10 mm KCl, 2 mm MgCl_2_, and 10% glycerol (pH 8). After extensive washing with the same buffer supplemented with 3 mm ATP and 10 mm MgCl_2_, bound proteins were eluted using 50 mm HEPES, 100 mm NaCl, 50 mm KCl, 10% glycerol, and 3 mm desthiobiotin (pH 7.5). Eluted proteins were either used directly or further purified by size‐exclusion chromatography on a Superdex 16/600 HR200 column (Cytiva, Marlborough, MA, USA) in 30 mm HEPES, 140 mm NaCl, 10 mm KCl, 3% glycerol, and 0.25 mm TCEP (pH 7.4). Protein purity was assessed by SDS/PAGE, and final preparations were concentrated, flash‐frozen in liquid nitrogen, and stored at −80 °C.

### Expression and purification of SIRT2

Human SIRT2 (amino acids 52–356; Uniprot Q8IXJ6) was expressed in *E. coli* Rosetta(DE3) cells from a modified pEC566‐SIRT2 plasmid as a fusion with an N‐terminal His_6_‐MBP tag (Fig. S10). Cells were cultured at 37 °C in LB medium supplemented with 100 μg·ml^−1^ ampicillin and 5 μm zinc acetate. At OD_600_ of ~ 0.5, expression was induced by 0.2 mm IPTG and cultivation continued at 18 °C for 20 h. Harvested cells were resuspended in 50 mm Tris–HCl, 300 mm NaCl, 10% glycerol, 20 mm imidazole, pH 7.8, and supplemented with the protease inhibitor cocktail (Roche). Cells were disintegrated by sonication and centrifuged for 30 min at 30 min/30 000 g/4 °C. Cleared lysate was loaded onto a Ni‐NTA column (IBA), thoroughly washed, and bound proteins eluted with 50 mm Tris–HCl, 300 mm NaCl, 0.4 m imidazole, 10% glycerol, pH 7.8. Elution fractions were concentrated and buffer exchanged to 50 mm Tris–HCl, 300 mm NaCl, 10% glycerol, 20 mm imidazole, pH 7.8, by dialysis overnight with simultaneous digestion of the fusion by TEV protease (substrate:enzyme w/w ratio of 50 : 1). The TEV protease and the cleaved His6‐MBP tag were removed by Ni‐NTA chromatography. The final purification step comprised size‐exclusion chromatography using a Superdex 75 16/600 column (Cytiva) equilibrated with 20 mm Tris–HCl, 150 mm NaCl, 10 mm KCl, pH 8. Purified SIRT2 was concentrated, flash‐frozen in liquid nitrogen and stored at −80 °C (Fig. S11).

### Expression and purification of SIRT1

Human SIRT1 (residues 143–747) was expressed in *E. coli* BL21 (RIPL) cells using a modified pESUMO‐SIRT1(143–747) plasmid encoding an N‐terminal His₆–SUMO fusion. Cells were grown in LB medium supplemented with 100 μg·ml^−1^ ampicillin and 0.2% glucose at 37 °C to an OD₆₀₀ of ~ 1. Cultures were cooled to 15 °C, induced with 0.5 mm IPTG, and incubated for 20 h at 15 °C. Cells were harvested by centrifugation (6000 ***g***, 15 min, 4 °C) and resuspended in lysis buffer (500 mm NaCl, 25 mm Tris–HCl, 1 mm β‐mercaptoethanol, pH 7.8) supplemented with EDTA‐free protease inhibitor cocktail (Roche). Cells were lysed by sonication (6 min total pulsing: 10 s on/30 s off; amplitude 7; probe 4420, Q700 sonicator, Qsonica) on ice. The lysate was clarified by two successive centrifugation steps (14 000 ***g***, 30 min, 4 °C; followed by 40 000 ***g***, 45 min, 4 °C). The supernatant was filtered and incubated with Ni‐NTA Superflow resin (IBA) for 1 h at 4 °C. The resin was washed thoroughly with lysis buffer (3 cycles), and bound protein was eluted with 80 mm imidazole in lysis buffer. The His₆–SUMO–SIRT1 fusion was further purified by size‐exclusion chromatography on a HiLoad Superdex 200 pg 10/300 column (Cytiva) equilibrated in 25 mm Tris–HCl, 300 mm NaCl, 1.5 mm DTT, pH 7.6. The purified protein was concentrated to 4.1 mg·ml^−1^, flash‐frozen in liquid nitrogen, and stored at −80 °C (Fig. S12).

### AcK‐CTTN deacetylation *in vitro*

*In vitro* deacetylation assays were performed in 50 mm HEPES, 140 mm NaCl, 10 mm KCl, 0.1% CHAPS, pH 7.4, buffer supplemented with 0.1 mm TCEP (HDACs 1–11) or 10 mm NAD^+^ (SIRT1 and SIRT2). In some experiments, the reaction mixtures further contained 0.2 mg·ml^−1^ BSA. Experiments were carried out at 37 °C and a stirring speed of 300 rpm (Thermomixer comfort, Eppendorf, Hamburg, Germany). AcK‐CTTN variants were used at 0.5 μm, and the substrate‐to‐enzyme molar ratio ranged between 10 : 1 and 1 : 1. At indicated time points, aliquots of reaction mixtures were collected, mixed with the SDS/PAGE sample buffer and heat‐denatured (95 °C, 5 min). Cortactin acetylation was analyzed by immunoblotting using the rabbit α‐AcK/goat α‐rabbit IgG HRP conjugate mixture (see below).

### Western blotting

Proteins were separated by SDS/PAGE and blotted on a PVDF membrane using a semidry Turbo blotter (Bio‐Rad). The membrane was blocked in 5% non‐fat dried milk dissolved in PBS‐T buffer (PBS buffer with 0.05% Tween 20) for 1 h and incubated with either (a) monoclonal α‐polyHis‐HRP antibody (Sigma‐Aldrich, A7058, dilution 1:20000), (b) StrepMAB‐Classic HRP conjugate (IBA, dilution 1 : 32000), or (c) rabbit α‐acetylated lysine antibody (α‐AcK antibody, Cell Signalling, 9441, dilution 1 : 1000)/goat α‐rabbit IgG HRP conjugate (Sigma‐Aldrich, A6154, 1 : 20000). All antibodies were diluted in 2% non‐fat dried milk in PBS‐T buffer. The incubation with the α‐AcK antibody was performed overnight at 4 °C, whereas the 1.5 h incubation at room temperature was used for other antibodies. After each incubation, the membrane was thoroughly washed with PBS‐T. Chemiluminescence detection was carried out using Immobilon Forte Western HRP substrate (Merck). Bands were visualized using an ImageQuant LAS4000 imager (Cytiva) and quantified with Quantity One 1‐D Analysis Software (Bio‐Rad, Hercules, CA, USA). Signal intensities of AcK‐CTTN bands were normalized to control reactions without HDAC, which were arbitrarily set to 100%.

### Deacetylation of AcK‐CTTN‐derived peptides

13‐mer peptides encompassing acetylation sites within the tandem repeats of human CTTN were synthesized commercially and their sequences are shown in Fig. 4A. An N‐terminal aminobenzoyl (Abz) group was incorporated to enable fluorescent detection during reverse‐phase HPLC (RP‐HPLC) analysis. Deacetylation assays were performed as described previously [11], with minor modifications. Briefly, peptides (0.8–400 μm) were incubated with optimized concentrations of HDACs in assay buffer containing 50 mm HEPES, 140 mm NaCl, 10 mm KCl, 0.1% bovine serum albumin (BSA), and 1 mm TCEP (pH 7.4), at 37 °C for 30 min. Reactions were quenched by adding 1/10 volume of 5% acetic acid and centrifuged at 2000 ***g***, 37 °C for 15 min to remove precipitated BSA and HDAC enzymes. Supernatants were analyzed by RP‐HPLC using a Shimadzu HPLC Prominence system equipped with a Kinetex 2.6 μm Polar C18 100 Å column (50 × 3 mm; Phenomenex, Torrance, CA, USA). Separation was achieved with a 12 min linear gradient from 10% to 35% of eluent B (95% acetonitrile, 0.1% TFA) at a flow rate of 0.6 ml ·min^−1^; eluent A consisted of 5% acetonitrile with 0.1% TFA. Substrates and reaction products were detected by fluorescence (excitation/emission: 320/420 nm). Product quantification was performed using a peptide standard curve, and kinetic parameters were derived by nonlinear regression analysis using GraphPad Prism (graphpad Software, San Diego, CA, USA).

### Peptide synthesis and analysis

Most Fmoc‐protected amino acids were purchased from GL Biochem Ltd (Shanghai, China). *N*,*N*‐Dimethylformamide (DMF), *N*,*N*′‐diisopropylcarbodiimide (DIC), ethyl cyanohydroxyiminoacetate (Oxyma) and Rink amide resin (0.65 mmol g^−1^) were purchased from Iris Biotech (Marktredwitz, Germany). (San Diego, USA). *N*,*N*‐Diisopropylethylamine (DIPEA), trifluoroacetic acid (TFA) and dichloromethane (DCM) were purchased from Carl Roth (Karlsruhe, Germany). Acetonitrile (ACN) was obtained from Avantor (Radnor, PA, USA). All other reagents were purchased from Sigma‐Aldrich / Merck KgaA (Darmstadt, Germany), unless stated otherwise.

Peptides were synthesized on Rink amide resin using Fmoc‐based solid‐phase peptide synthesis on an automated microwave peptide synthesizer Liberty Blue (CEM Corporation). Fmoc amino acid (5 eq) couplings were carried out with DIC (10 eq)/Oxyma (5 eq) at 90 °C for 2 min twice, except for 2‐(Boc‐amino)benzoic acid (Boc‐Abz‐OH), which was coupled twice for 30 min at room temperature. Fmoc deprotection was accomplished using a 10% (w/v) piperazine solution in a *N*‐methylpyrrolidone‐ethanol (9 : 1, v/v) mixture containing 0.1 m 1‐hydroxybenzotriazole at 90 °C for 1 min. All peptides were cleaved from the resin using TFA‐H_2_O (95 : 5, v/v) for 1 h, repeated twice. TFA was evaporated *in vacuo*, and the crude peptides were purified with preparative HPLC. Fractions containing the pure peptide were combined and lyophilized.

HPLC analysis was performed using a Waters ACQUITY UPLC‐MS system (Milford, USA) equipped with a Waters ACQUITY UPLC BEH C18 column (1.7 μm, 2.1 x 50 mm, 30 Å). As a mobile phase, 0.1% formic acid in H_2_O (solvent A) and 0.1% formic acid in ACN (solvent B) solutions were used. A typical gradient from 5 : 95 (v/v) of ACN/H_2_O to 95:5 (v/v) of ACN/H_2_O over 6 min was used for most runs. QTOF spectra were made on a Waters Q‐Tof Premier (Milford, MA, USA) mass spectrometer. Data were analyzed using Waters MassLynx software (Fig. S13). Peptide purification was carried out on a Shimadzu LC System with a Lichrospher® 100 RP‐8 column (5 μm, 250 × 25 mm) with gradients of 0.05% TFA in H_2_O (solvent A) and 0.05% TFA in ACN (solvent B).

## Authors contribution

Conceptualization: JK, CB Methodology: JK, MV, ZK, ZN, JKu, PJ, JP, RT, BH, JK, CS‐F, MM. Formal Analysis: JK, MV, ZK, JKu, RT, PJ, JP, BH. Visualization: JK, MV, JKu, RT, MM. Writing – Original Draft: JK, CB. Writing – Review & Editing: All authors. Resources: CS‐F, MM, MS. Funding Acquisition: JK, CB.

## Declaration of generative AI and AI‐assisted technologies in the writing process

During the preparation of this work the author(s) used CHATGPT in order to improve the readability and language of the manuscript. After using this tool/service, the author(s) reviewed and edited the content as needed and take full responsibility for the content of the published article.

## Conflict of interest

The authors declare no conflict of interest.

## Supporting information


**Table S1.** Concentrations of substrates and HDACs used for analysis of deacetylation activity by RP‐HPLC.
**Table S2**. Primers used for generation of site‐specifically acetylated cortactin variants.
**Fig. S1**. The plasmid map and the amino acid sequence of the wild‐type cortactin expression construct.
**Fig. S2**. MS/MS analysis of tryptic cortactin peptides.
**Fig. S3**. Replicates of Western blots of deacetylation reactions of AcK‐CTTN variants by human HDACs 1–11 (Fig. 2 of the main text).
**Fig. S4**. Replicates of Western blots of deacetylation reactions of AcK‐CTTN variants by human HDAC6, HDAC8, SIRT1, and SIRT2 (Fig. 3 of the main text).
**Fig. S5**. Replicates of Western blots of deacetylation reactions by human SIRT2 (Fig. 4 of the main text).
**Fig. S6**. The Michaelis‐Menten plot of deacetylation of AcK‐CTTN‐derived peptides by wild‐type HDAC6.
**Fig. S7**. Deacetylation of substrate‐derived peptides by selected HDACs.
**Fig. S8**. Uncropped Western blots of deacetylation reactions of AcK‐CTTN by HDAC6 variants.
**Fig. S9**. Uncropped Western blots of expression of wild‐type and AcK‐CTTN variants in *E.coli*.
**Fig. S10**. The plasmid map and the amino acid sequence of the SIRT2 expression construct.
**Fig. S11**. Purification of human SIRT2.
**Fig. S12**. Purification of human SIRT1.
**Fig. S13**. QTOF‐MS (ESI+) spectra of H3K9 and p53AcK382 peptides as class I HDAC substrates.

## Data Availability

The data that support the findings of this study are available from the corresponding author (cyril.barinka@ibt.cas.cz) upon reasonable request.

## References

[1] Ali I , Conrad RJ , Verdin E & Ott M (2018) Lysine acetylation goes global: from epigenetics to metabolism and therapeutics. Chem Rev 118, 1216–1252. 29405707 10.1021/acs.chemrev.7b00181 PMC6609103

[2] Escalante‐Semerena JC (2010) N ε‐lysine acetylation control conserved in all three life domains: the relative simplicity of studying microbes could prove critical for understanding this posttranslational modification system. Microbe Wash DC 5, 340–344. 33907535 10.1128/microbe.5.340.1 PMC8075172

[3] Narita T , Weinert BT & Choudhary C (2019) Functions and mechanisms of non‐histone protein acetylation. Nat Rev Mol Cell Biol 20, 156–174. 30467427 10.1038/s41580-018-0081-3

[4] Bali P , Pranpat M , Bradner J , Balasis M , Fiskus W , Guo F , Rocha K , Kumaraswamy S , Boyapalle S , Atadja P *et al*. (2005) Inhibition of histone deacetylase 6 acetylates and disrupts the chaperone function of heat shock protein 90: a novel basis for antileukemia activity of histone deacetylase inhibitors. J Biol Chem 280, 26729–26734. 15937340 10.1074/jbc.C500186200

[5] Barber MF , Michishita‐Kioi E , Xi Y , Tasselli L , Kioi M , Moqtaderi Z , Tennen RI , Paredes S , Young NL , Chen K *et al*. (2012) SIRT7 links H3K18 deacetylation to maintenance of oncogenic transformation. Nature 487, 114–118. 22722849 10.1038/nature11043 PMC3412143

[6] Hubbert C , Guardiola A , Shao R , Kawaguchi Y , Ito A , Nixon A , Yoshida M , Wang X‐F & Yao T‐P (2002) HDAC6 is a microtubule‐associated deacetylase. Nature 417, 455–458. 12024216 10.1038/417455a

[7] Knyphausen P , de Boor S , Kuhlmann N , Scislowski L , Extra A , Baldus L , Schacherl M , Baumann U , Neundorf I & Lammers M (2016) Insights into lysine deacetylation of natively folded substrate proteins by Sirtuins. J Biol Chem 291, 14677–14694. 27226597 10.1074/jbc.M116.726307 PMC4938187

[8] Kovacs JJ , Murphy PJM , Gaillard S , Zhao X , Wu J‐T , Nicchitta CV , Yoshida M , Toft DO , Pratt WB & Yao T‐P (2005) HDAC6 regulates Hsp90 acetylation and chaperone‐dependent activation of glucocorticoid receptor. Mol Cell 18, 601–607. 15916966 10.1016/j.molcel.2005.04.021

[9] Luo J , Su F , Chen D , Shiloh A & Gu W (2000) Deacetylation of p53 modulates its effect on cell growth and apoptosis. Nature 408, 377–381. 11099047 10.1038/35042612

[10] Matsuyama A , Shimazu T , Sumida Y , Saito A , Yoshimatsu Y , Seigneurin‐Berny D , Osada H , Komatsu Y , Nishino N , Khochbin S *et al*. (2002) *In vivo* destabilization of dynamic microtubules by HDAC6‐mediated deacetylation. EMBO J 21, 6820–6831. 12486003 10.1093/emboj/cdf682 PMC139102

[11] Ustinova K , Novakova Z , Saito M , Meleshin M , Mikesova J , Kutil Z , Baranova P , Havlinova B , Schutkowski M , Matthias P *et al*. (2020) The disordered N‐terminus of HDAC6 is a microtubule‐binding domain critical for efficient tubulin deacetylation. J Biol Chem 295, 2614–2628. 31953325 10.1074/jbc.RA119.011243 PMC7049964

[12] Vaquero A , Scher M , Lee D , Erdjument‐Bromage H , Tempst P & Reinberg D (2004) Human SirT1 interacts with histone H1 and promotes formation of facultative heterochromatin. Mol Cell 16, 93–105. 15469825 10.1016/j.molcel.2004.08.031

[13] Vaquero A , Scher MB , Lee DH , Sutton A , Cheng H‐L , Alt FW , Serrano L , Sternglanz R & Reinberg D (2006) SirT2 is a histone deacetylase with preference for histone H4 Lys 16 during mitosis. Genes Dev 20, 1256–1261. 16648462 10.1101/gad.1412706 PMC1472900

[14] Vaziri H , Dessain SK , Ng Eaton E , Imai SI , Frye RA , Pandita TK , Guarente L & Weinberg RA (2001) hSIR2(SIRT1) functions as an NAD‐dependent p53 deacetylase. Cell 107, 149–159. 11672523 10.1016/s0092-8674(01)00527-x

[15] Vermeulen M , Carrozza MJ , Lasonder E , Workman JL , Logie C & Stunnenberg HG (2004) *In vitro* targeting reveals intrinsic histone tail specificity of the Sin3/histone deacetylase and N‐CoR/SMRT corepressor complexes. Mol Cell Biol 24, 2364–2372. 14993276 10.1128/MCB.24.6.2364-2372.2004 PMC355843

[16] Zhang Y , Li N , Caron C , Matthias G , Hess D , Khochbin S & Matthias P (2003) HDAC‐6 interacts with and deacetylates tubulin and microtubules *in vivo* . EMBO J 22, 1168–1179. 12606581 10.1093/emboj/cdg115 PMC150348

[17] Zhao Z , Xu H & Gong W (2010) Histone deacetylase 6 (HDAC6) is an independent deacetylase for alpha‐tubulin. Protein Pept Lett 17, 555–558. 19961433 10.2174/092986610791112620

[18] Kaypee S , Sudarshan D , Shanmugam MK , Mukherjee D , Sethi G & Kundu TK (2016) Aberrant lysine acetylation in tumorigenesis: implications in the development of therapeutics. Pharmacol Ther 162, 98–119. 26808162 10.1016/j.pharmthera.2016.01.011

[19] Selvi RB & Kundu TK (2009) Reversible acetylation of chromatin: implication in regulation of gene expression, disease and therapeutics. Biotechnol J 4, 375–390. 19296442 10.1002/biot.200900032

[20] Wang X , Wei X , Pang Q & Yi F (2012) Histone deacetylases and their inhibitors: molecular mechanisms and therapeutic implications in diabetes mellitus. Acta Pharm Sin B 2, 387–395.

[21] Yang M , Zhang Y & Ren J (2020) Acetylation in cardiovascular diseases: molecular mechanisms and clinical implications. Biochim Biophys Acta Mol basis Dis 1866, 165836. 32413386 10.1016/j.bbadis.2020.165836

[22] Kanner SB , Reynolds AB , Vines RR & Parsons JT (1990) Monoclonal antibodies to individual tyrosine‐phosphorylated protein substrates of oncogene‐encoded tyrosine kinases. Proc Natl Acad Sci USA 87, 3328–3332. 2110361 10.1073/pnas.87.9.3328 PMC53893

[23] Wu H , Reynolds AB , Kanner SB , Vines RR & Parsons JT (1991) Identification and characterization of a novel cytoskeleton‐associated pp60src substrate. Mol Cell Biol 11, 5113–5124. 1922035 10.1128/mcb.11.10.5113-5124.1991 PMC361526

[24] Kaksonen M , Peng HB & Rauvala H (2000) Association of cortactin with dynamic actin in lamellipodia and on endosomal vesicles. J Cell Sci 113, 4421–4426. 11082035 10.1242/jcs.113.24.4421

[25] Weed SA , Karginov AV , Schafer DA , Weaver AM , Kinley AW , Cooper JA & Parsons JT (2000) Cortactin localization to sites of actin assembly in lamellipodia requires interactions with F‐actin and the Arp2/3 complex. J Cell Biol 151, 29–40. 11018051 10.1083/jcb.151.1.29 PMC2189811

[26] Wu H & Parsons JT (1993) Cortactin, an 80/85‐kilodalton pp60src substrate, is a filamentous actin‐binding protein enriched in the cell cortex. J Cell Biol 120, 1417–1426. 7680654 10.1083/jcb.120.6.1417 PMC2119758

[27] MacGrath SM & Koleske AJ (2012) Cortactin in cell migration and cancer at a glance. J Cell Sci 125, 1621–1626. 22566665 10.1242/jcs.093781 PMC3346822

[28] Yin M , Ma W & An L (2017) Cortactin in cancer cell migration and invasion. Oncotarget 8, 88232–88243. 29152154 10.18632/oncotarget.21088 PMC5675706

[29] Uruno T , Liu J , Zhang P , Fan Yx n , Egile C , Li R , Mueller SC & Zhan X (2001) Activation of Arp2/3 complex‐mediated actin polymerization by cortactin. Nat Cell Biol 3, 259–266. 11231575 10.1038/35060051

[30] Kirkbride KC , Sung BH , Sinha S & Weaver AM (2011) Cortactin: a multifunctional regulator of cellular invasiveness. Cell Adhes Migr 5, 187–198. 10.4161/cam.5.2.14773 PMC3084985 21258212

[31] Huang C , Liu J , Haudenschild CC & Zhan X (1998) The role of tyrosine phosphorylation of cortactin in the locomotion of endothelial cells. J Biol Chem 273, 25770–25776. 9748248 10.1074/jbc.273.40.25770

[32] Castro‐Castro A , Janke C , Montagnac G , Paul‐Gilloteaux P & Chavrier P (2012) ATAT1/MEC‐17 acetyltransferase and HDAC6 deacetylase control a balance of acetylation of alpha‐tubulin and cortactin and regulate MT1‐MMP trafficking and breast tumor cell invasion. Eur J Cell Biol 91, 950–960. 22902175 10.1016/j.ejcb.2012.07.001

[33] Sun Y , Sun J , Lungchukiet P , Quarni W , Yang S , Zhang X & Bai W (2015) Fe65 suppresses breast cancer cell migration and invasion through Tip60 mediated cortactin acetylation. Sci Rep 5, 11529. 26166158 10.1038/srep11529 PMC4499803

[34] Zhang X , Yuan Z , Zhang Y , Yong S , Salas‐Burgos A , Koomen J , Olashaw N , Parsons JT , Yang X‐J , Dent SR *et al*. (2007) HDAC6 modulates cell motility by altering the acetylation level of cortactin. Mol Cell 27, 197–213. 17643370 10.1016/j.molcel.2007.05.033 PMC2684874

[35] Zhang Y , Zhang M , Dong H , Yong S , Li X , Olashaw N , Kruk PA , Cheng JQ , Bai W , Chen J *et al*. (2009) Deacetylation of cortactin by SIRT1 promotes cell migration. Oncogene 28, 445–460. 18850005 10.1038/onc.2008.388

[36] Ma J , Zhang L‐Q , He Z‐X , He X‐X , Wang Y‐J , Jian Y‐L , Wang X , Zhang B‐B , Su C , Lu J *et al*. (2019) Autism candidate gene DIP2A regulates spine morphogenesis via acetylation of cortactin. PLoS Biol 17, e3000461. 31600191 10.1371/journal.pbio.3000461 PMC6786517

[37] Choudhary C , Kumar C , Gnad F , Nielsen ML , Rehman M , Walther TC , Olsen JV & Mann M (2009) Lysine acetylation targets protein complexes and co‐regulates major cellular functions. Science 325, 834–840. 19608861 10.1126/science.1175371

[38] Hornbeck PV , Zhang B , Murray B , Kornhauser JM , Latham V & Skrzypek E (2015) PhosphoSitePlus, 2014: mutations, PTMs and recalibrations. Nucleic Acids Res 43, D512–D520. 25514926 10.1093/nar/gku1267 PMC4383998

[39] Olson DE , Udeshi ND , Wolfson NA , Pitcairn CA , Sullivan ED , Jaffe JD , Svinkina T , Natoli T , Lu X , Paulk J *et al*. (2014) An unbiased approach to identify endogenous substrates of histone deacetylase 8. ACS Chem Biol 9, 2210–2216. 25089360 10.1021/cb500492r PMC4201337

[40] Park J , Chen Y , Tishkoff DX , Peng C , Tan M , Dai L , Xie Z , Zhang Y , Zwaans BMM , Skinner ME *et al*. (2013) SIRT5‐mediated lysine desuccinylation impacts diverse metabolic pathways. Mol Cell 50, 919–930. 23806337 10.1016/j.molcel.2013.06.001 PMC3769971

[41] Schölz C , Weinert BT , Wagner SA , Beli P , Miyake Y , Qi J , Jensen LJ , Streicher W , McCarthy AR , Westwood NJ *et al*. (2015) Acetylation site specificities of lysine deacetylase inhibitors in human cells. Nat Biotechnol 33, 415–423. 25751058 10.1038/nbt.3130

[42] Weinert BT , Schölz C , Wagner SA , Iesmantavicius V , Su D , Daniel JA & Choudhary C (2013) Lysine succinylation is a frequently occurring modification in prokaryotes and eukaryotes and extensively overlaps with acetylation. Cell Rep 4, 842–851. 23954790 10.1016/j.celrep.2013.07.024

[43] Huang Y , Zhai G , Fu Y , Li Y , Zang Y , Lin Y & Zhang K (2024) A proximity labeling‐based orthogonal trap strategy identifies HDAC8 promotes cell motility by modulating cortactin acetylation. Cell Chem Biol 31, 514–522. 38460516 10.1016/j.chembiol.2024.02.003

[44] Hsieh Y‐L , Tu H‐J , Pan S‐L , Liou J‐P & Yang C‐R (2019) Anti‐metastatic activity of MPT0G211, a novel HDAC6 inhibitor, in human breast cancer cells *in vitro* and *in vivo* . Biochim Biophys Acta Mol Cell Res 1866, 992–1003. 30867138 10.1016/j.bbamcr.2019.03.003

[45] Kaluza D , Kroll J , Gesierich S , Yao T‐P , Boon RA , Hergenreider E , Tjwa M , Rössig L , Seto E , Augustin HG *et al*. (2011) Class IIb HDAC6 regulates endothelial cell migration and angiogenesis by deacetylation of cortactin. EMBO J 30, 4142–4156. 21847094 10.1038/emboj.2011.298 PMC3199386

[46] Kim J‐Y , Hwang H‐G , Lee J‐Y , Kim M & Kim J‐Y (2020) Cortactin deacetylation by HDAC6 and SIRT2 regulates neuronal migration and dendrite morphogenesis during cerebral cortex development. Mol Brain 13, 105. 32711564 10.1186/s13041-020-00644-y PMC7382832

[47] Messaoudi K , Ali A , Ishaq R , Palazzo A , Sliwa D , Bluteau O , Souquère S , Muller D , Diop KM , Rameau P *et al*. (2017) Critical role of the HDAC6‐cortactin axis in human megakaryocyte maturation leading to a proplatelet‐formation defect. Nat Commun 8, 1786. 29176689 10.1038/s41467-017-01690-2 PMC5702605

[48] Motonishi S , Nangaku M , Wada T , Ishimoto Y , Ohse T , Matsusaka T , Kubota N , Shimizu A , Kadowaki T , Tobe K *et al*. (2015) Sirtuin1 maintains actin cytoskeleton by deacetylation of cortactin in injured podocytes. J Am Soc Nephrol 26, 1939–1959. 25424328 10.1681/ASN.2014030289 PMC4520160

[49] Nakane K , Fujita Y , Terazawa R , Atsumi Y , Kato T , Nozawa Y , Deguchi T & Ito M (2012) Inhibition of cortactin and SIRT1 expression attenuates migration and invasion of prostate cancer DU145 cells. Int J Urol 19, 71–79. 22050448 10.1111/j.1442-2042.2011.02888.x

[50] Ran J , Yang Y , Li D , Liu M & Zhou J (2015) Deacetylation of α‐tubulin and cortactin is required for HDAC6 to trigger ciliary disassembly. Sci Rep 5, 12917. 26246421 10.1038/srep12917 PMC4526867

[51] Rey M , Irondelle M , Waharte F , Lizarraga F & Chavrier P (2011) HDAC6 is required for invadopodia activity and invasion by breast tumor cells. Eur J Cell Biol 90, 128–135. 20970878 10.1016/j.ejcb.2010.09.004

[52] Saito M , Hess D , Eglinger J , Fritsch AW , Kreysing M , Weinert BT , Choudhary C & Matthias P (2019) Acetylation of intrinsically disordered regions regulates phase separation. Nat Chem Biol 15, 51–61. 30531905 10.1038/s41589-018-0180-7

[53] Tsunoda K , Oikawa H , Tada H , Tatemichi Y , Muraoka S , Miura S , Shibazaki M , Maeda F , Takahashi K , Akasaka T *et al*. (2011) Nucleus accumbens‐associated 1 contributes to cortactin deacetylation and augments the migration of melanoma cells. J Invest Dermatol 131, 1710–1719. 21562571 10.1038/jid.2011.110

[54] Lin Y , Liu Q , Li L , Yang R , Ye J , Yang S , Luo G , Reinach PS & Yan D (2022) Sirt1 regulates corneal epithelial migration by deacetylating cortactin. Invest Ophthalmol Vis Sci 63, 14. 10.1167/iovs.63.12.14 PMC9652720 36350618

[55] Iwahara N , Azekami K , Hosoda R , Nojima I , Hisahara S & Kuno A (2022) Activation of SIRT1 promotes membrane resealing via cortactin. Sci Rep 12, 15328. 36097021 10.1038/s41598-022-19136-1 PMC9468153

[56] Li J , Chen S , Cleary RA , Wang R , Gannon OJ , Seto E & Tang DD (2014) Histone deacetylase 8 regulates cortactin deacetylation and contraction in smooth muscle tissues. Am J Physiol Cell Physiol 307, C288–C295. 24920679 10.1152/ajpcell.00102.2014 PMC4121581

[57] Zhang Y , Zou J , Tolbert E , Zhao TC , Bayliss G & Zhuang S (2020) Identification of histone deacetylase 8 as a novel therapeutic target for renal fibrosis. FASEB J 34, 7295–7310. 32281211 10.1096/fj.201903254R PMC7445474

[58] Weaver AM , Heuser JE , Karginov AV , Lee W‐l , Parsons JT & Cooper JA (2002) Interaction of cortactin and N‐WASp with Arp2/3 complex. Curr Biol 12, 1270–1278. 12176354 10.1016/s0960-9822(02)01035-7

[59] Neumann H , Peak‐Chew SY & Chin JW (2008) Genetically encoding N(epsilon)‐acetyllysine in recombinant proteins. Nat Chem Biol 4, 232–234. 18278036 10.1038/nchembio.73

[60] Chatterjee A , Sun SB , Furman JL , Xiao H & Schultz PG (2013) A versatile platform for single‐ and multiple‐unnatural amino acid mutagenesis in Escherichia coli. Biochemistry 52, 1828–1837. 23379331 10.1021/bi4000244 PMC3855549

[61] Kutil Z , Meleshin M , Baranova P , Havlinova B , Schutkowski M & Barinka C (2022) Characterization of the class IIa histone deacetylases substrate specificity. FASEB J 36, e22287. 35349187 10.1096/fj.202101663R

[62] Kutil Z , Novakova Z , Meleshin M , Mikesova J , Schutkowski M & Barinka C (2018) Histone deacetylase 11 is a fatty‐acid deacylase. ACS Chem Biol 13, 685–693. 29336543 10.1021/acschembio.7b00942

[63] Kutil Z , Skultetyova L , Rauh D , Meleshin M , Snajdr I , Novakova Z , Mikesova J , Pavlicek J , Hadzima M , Baranova P *et al*. (2019) The unraveling of substrate specificity of histone deacetylase 6 domains using acetylome peptide microarrays and peptide libraries. FASEB J 33, 4035–4045. 30496698 10.1096/fj.201801680R

[64] Ptacek J , Snajdr I , Schimer J , Kutil Z , Mikesova J , Baranova P , Havlinova B , Tueckmantel W , Majer P , Kozikowski A *et al*. (2023) Selectivity of hydroxamate‐ and Difluoromethyloxadiazole‐based inhibitors of histone deacetylase 6 *in vitro* and in cells. Int J Mol Sci 24, 4720. 36902164 10.3390/ijms24054720 PMC10003107

[65] Guenther MG , Barak O & Lazar MA (2001) The SMRT and N‐CoR corepressors are activating cofactors for histone deacetylase 3. Mol Cell Biol 21, 6091–6101. 11509652 10.1128/MCB.21.18.6091-6101.2001 PMC87326

[66] Wen YD , Perissi V , Staszewski LM , Yang WM , Krones A , Glass CK , Rosenfeld MG & Seto E (2000) The histone deacetylase‐3 complex contains nuclear receptor corepressors. Proc Natl Acad Sci USA 97, 7202–7207. 10860984 10.1073/pnas.97.13.7202 PMC16523

[67] Gomes ID , Ariyaratne UV & Pflum MKH (2021) HDAC6 substrate discovery using proteomics‐based substrate trapping: HDAC6 deacetylates PRMT5 to influence methyltransferase activity. ACS Chem Biol 16, 1435–1444. 34314149 10.1021/acschembio.1c00303 PMC8939004

[68] Hai Y , Shinsky SA , Porter NJ & Christianson DW (2017) Histone deacetylase 10 structure and molecular function as a polyamine deacetylase. Nat Commun 8, 15368. 28516954 10.1038/ncomms15368 PMC5454378

[69] Seto E & Yoshida M (2014) Erasers of histone acetylation: the histone deacetylase enzymes. Cold Spring Harb Perspect Biol 6, a018713. 24691964 10.1101/cshperspect.a018713 PMC3970420

[70] Gurard‐Levin ZA & Mrksich M (2008) The activity of HDAC8 depends on local and distal sequences of its peptide substrates. Biochemistry 47, 6242–6250. 18470998 10.1021/bi800053v PMC2605276

[71] Cohen S , Kramarski L , Levi S , Deshe N , Ben David O & Arbely E (2019) Nonsense mutation‐dependent reinitiation of translation in mammalian cells. Nucleic Acids Res 47, 6330–6338. 31045216 10.1093/nar/gkz319 PMC6614817

[72] Gunišová S , Hronová V , Mohammad MP , Hinnebusch AG & Valášek LS (2018) Please do not recycle! translation reinitiation in microbes and higher eukaryotes. FEMS Microbiol Rev 42, 165–192. 29281028 10.1093/femsre/fux059 PMC5972666

[73] Kalstrup T & Blunck R (2015) Reinitiation at non‐canonical start codons leads to leak expression when incorporating unnatural amino acids. Sci Rep 5, 11866. 26153354 10.1038/srep11866 PMC4648390

[74] Chemla Y , Peeri M , Heltberg ML , Eichler J , Jensen MH , Tuller T & Alfonta L (2020) A possible universal role for mRNA secondary structure in bacterial translation revealed using a synthetic operon. Nat Commun 11, 4827. 32973167 10.1038/s41467-020-18577-4 PMC7518266

[75] Grozinger CM , Hassig CA & Schreiber SL (1999) Three proteins define a class of human histone deacetylases related to yeast Hda1p. Proc Natl Acad Sci USA 96, 4868–4873. 10220385 10.1073/pnas.96.9.4868 PMC21783

[76] Haggarty SJ , Koeller KM , Wong JC , Grozinger CM & Schreiber SL (2003) Domain‐selective small‐molecule inhibitor of histone deacetylase 6 (HDAC6)‐mediated tubulin deacetylation. Proc Natl Acad Sci USA 100, 4389–4394. 12677000 10.1073/pnas.0430973100 PMC153564

[77] Zhang Y , Gilquin B , Khochbin S & Matthias P (2006) Two catalytic domains are required for protein deacetylation *. J Biol Chem 281, 2401–2404. 16272578 10.1074/jbc.C500241200

[78] Zou H , Wu Y , Navre M & Sang B‐C (2006) Characterization of the two catalytic domains in histone deacetylase 6. Biochem Biophys Res Commun 341, 45–50. 16412385 10.1016/j.bbrc.2005.12.144

[79] Hai Y & Christianson DW (2016) Histone deacetylase 6 structure and molecular basis of catalysis and inhibition. Nat Chem Biol 12, 741–747. 27454933 10.1038/nchembio.2134 PMC4990478

[80] Toro TB & Watt TJ (2020) Critical review of non‐histone human substrates of metal‐dependent lysine deacetylases. FASEB J 34, 13140–13155. 32862458 10.1096/fj.202001301RR PMC7719617

[81] Zuo Q , Wu W , Li X , Zhao L & Chen W (2012) HDAC6 and SIRT2 promote bladder cancer cell migration and invasion by targeting cortactin. Oncol Rep 27, 819–824. 22089141 10.3892/or.2011.1553

[82] Nahhas F , Dryden SC , Abrams J & Tainsky MA (2007) Mutations in SIRT2 deacetylase which regulate enzymatic activity but not its interaction with HDAC6 and tubulin. Mol Cell Biochem 303, 221–230. 17516032 10.1007/s11010-007-9478-6

[83] North BJ , Marshall BL , Borra MT , Denu JM & Verdin E (2003) The human Sir2 ortholog, SIRT2, is an NAD+‐dependent tubulin deacetylase. Mol Cell 11, 437–444. 12620231 10.1016/s1097-2765(03)00038-8

[84] Di Sante G , Wang L , Wang C , Jiao X , Casimiro MC , Chen K , Pestell TG , Yaman I , Di Rocco A , Sun X *et al*. (2015) Sirt1‐deficient mice have hypogonadotropic hypogonadism due to defective GnRH neuronal migration. Mol Endocrinol 29, 200–212. 25545407 10.1210/me.2014-1228 PMC4318884

[85] Ito A , Shimazu T , Maeda S , Shah AA , Tsunoda T , Iemura S , Natsume T , Suzuki T , Motohashi H , Yamamoto M *et al*. (2015) The subcellular localization and activity of cortactin is regulated by acetylation and interaction with Keap1. Sci Signal 8, ra120. 26602019 10.1126/scisignal.aad0667

[86] Meiler E , Nieto‐Pelegrín E & Martinez‐Quiles N (2012) Cortactin tyrosine phosphorylation promotes its deacetylation and inhibits cell spreading. PLoS One 7, e33662. 22479425 10.1371/journal.pone.0033662 PMC3316595

[87] Shentu T‐P , He M , Sun X , Zhang J , Zhang F , Gongol B , Marin TL , Zhang J , Wen L , Wang Y *et al*. (2016) AMP‐activated protein kinase and Sirtuin 1 coregulation of cortactin contributes to endothelial function. Arterioscler Thromb Vasc Biol 36, 2358–2368. 27758765 10.1161/ATVBAHA.116.307871 PMC5391843

[88] Arbely E , Natan E , Brandt T , Allen MD , Veprintsev DB , Robinson CV , Chin JW , Joerger AC & Fersht AR (2011) Acetylation of lysine 120 of p53 endows DNA‐binding specificity at effective physiological salt concentration. Proc Natl Acad Sci USA 108, 8251–8256. 21525412 10.1073/pnas.1105028108 PMC3100949

[89] Philo JS (2023) SEDNTERP: a calculation and database utility to aid interpretation of analytical ultracentrifugation and light scattering data. Eur Biophys J 52, 233–266. 36792822 10.1007/s00249-023-01629-0

[90] Schuck P (2000) Size‐distribution analysis of macromolecules by sedimentation velocity ultracentrifugation and lamm equation modeling. Biophys J 78, 1606–1619. 10692345 10.1016/S0006-3495(00)76713-0 PMC1300758

[91] Brautigam CA (2015) Calculations and publication‐quality illustrations for analytical ultracentrifugation data. Methods Enzymol 562, 109–133. 26412649 10.1016/bs.mie.2015.05.001

[92] Skultetyova L , Ustinova K , Kutil Z , Novakova Z , Pavlicek J , Mikesova J , Trapl D , Baranova P , Havlinova B , Hubalek M *et al*. (2017) Human histone deacetylase 6 shows strong preference for tubulin dimers over assembled microtubules. Sci Rep 7, 11547. 28912522 10.1038/s41598-017-11739-3 PMC5599508

