# Supplementary material for: Selective targeting of cortactin tandem repeat acetylation by human lysine deacetylases
Source: FEBS J. 2026 Feb 4;293(12):3528–44. doi: 10.1111/febs.70430 (PMC13278358; doi:10.1111/febs.70430)
Supplement: Supplementary file 1 — Table S1. Concentrations of substrates and HDACs used for analysis of deacetylation activity by RP‐HPLC. Table S2. Primers used for generation of site‐specifically acetylated cortactin variants. Fig. S1. The plasmid map and the amino acid sequence of the wild‐type cortactin expression construct. Fig. S2. MS/MS analysis of tryptic cortactin peptides. Fig. S3. Replicates of Western blots of deacetylation reactions of AcK‐CTTN variants by human HDACs 1–11 (Fig. 2 of the main text). Fig. S4. Replicates of Western blots of deacetylation reactions of AcK‐CTTN variants by human HDAC6, HDAC8, SIRT1, and SIRT2 (Fig. 3 of the main text). Fig. S5. Replicates of Western blots of deacetylation reactions by human SIRT2 (Fig. 4 of the main text). Fig. S6. The Michaelis‐Menten plot of deacetylation of AcK‐CTTN‐derived peptides by wild‐type HDAC6. Fig. S7. Deacetylation of substrate‐derived peptides by selected HDACs. Fig. S8. Uncropped Western blots of deacetylation reactions of AcK‐CTTN by HDAC6 variants. Fig. S9. Uncropped Western blots of expression of wild‐type and AcK‐CTTN variants in E.coli. Fig. S10. The plasmid map and the amino acid sequence of the SIRT2 expression construct. Fig. S11. Purification of human SIRT2. Fig. S12. Purification of human SIRT1. Fig. S13. QTOF‐MS (ESI+) spectra of H3K9 and p53AcK382 peptides as class I HDAC substrates. [file FEBS-293-3528-s001.docx]

**SUPPORTING INFORMATION**

**Selective targeting of cortactin tandem repeat acetylation by human lysine deacetylases**

**Jan Komarek^1#^*, Miroslava Vosahlikova^1^, Zsofia Kutil^1^, Zora Novakova^1^, Julia Kudlacova^1^, Ruzena Tuckova^1^, Marat Meleshin^2^, Barbora Havlinova^1^, Pavlina Jaklova^1^, Jana Ptackova^1^, Cordelia Schiene-Fischer^2^, Mike Schutkowski^2^, Cyril Barinka^1^***

*^1^Institute of Biotechnology of the Czech Academy of Sciences, BIOCEV, Prumyslova 595, 252 50 Vestec, Czech Republic*

*^2^Department of Enzymology, Charles Tanford Protein Center, Institute of Biochemistry and Biotechnology, Martin-Luther-University Halle-Wittenberg, Halle/Saale (Germany)*

**Supplementary Tables and Figures:**

**List of Tables:**

**Table S1:** Concentrations of substrates and HDACs used for analysis of deacetylation activity by RP-HPLC

**Table S2:** Primers used for generation of site-specifically acetylated cortactin variants

**List of Figures:**

**Figure S1:** The plasmid map and the amino acid sequence of the wild-type cortactin expression construct.

**Figure S2:** MS/MS analysis of tryptic cortactin peptides.

**Figure S3:** Replicates of Western blots of deacetylation reactions of AcK-CTTN variants by human HDACs 1–11 (Fig. 2 of the main text).

**Figure S4:** Replicates of Western blots of deacetylation reactions of AcK-CTTN variants by human HDAC6, HDAC8, SIRT1, and SIRT2 (Fig. 3 of the main text).

**Figure S5:** Replicates of Western blots of deacetylation reactions by human SIRT2 (Fig. 4 of the main text).

**Figure S6:** The Michaelis-Menten plot of deacetylation of AcK-CTTN-derived peptides by wild-type HDAC6.

**Figure S7**: Deacetylation of substrate-derived peptides by selected HDACs.

**Figure S8:** Uncropped Western blots of deacetylation reactions of AcK-CTTN by HDAC6 variants.

**Figure S9:** Uncropped Western blots of expression of wild-type and AcK-CTTN variants in *E. coli*.

**Figure S10:** The plasmid map and the amino acid sequence of the SIRT2 expression construct.

**Figure S11:** Purification of human SIRT2.

**Figure S12:** Purification of human SIRT1.

**Figure S13:** QTOF-MS (ESI+) spectra of H3K9 and p53AcK382 peptides as class I HDAC substrates.

**Tables:**

**Table S1:** Concentrations of substrates and HDACs used for analysis of deacetylation activity by RP-HPLC.

| **Name** | **Peptide** | ***c_peptide_* (μM)** | ***c_protein_* (nM)** |
| --- | --- | --- | --- |
| AcK87 | Abz-SHGYGGK(Ac)FGVEQD-NH_2_ | 20 | 50 |
| AcK124 | Abz-VRGFGGK(Ac)FGVQMD-NH_2_ | 20 | 10 |
| AcK161 | Abz-SSGFGGK(Ac)YGVQAD-NH_2_ | 20 | 25 |
| AcK198 | Abz-SKGFGGK(Ac)YGIDKD-NH_2_ | 20 | 8 |
| AcK235 | Abz-VKGFGGK(Ac)FGVQTD-NH_2_ | 20 | 30 |
| AcK272 | Abz-KTGFGGK(Ac)FGVQSE-NH_2_ | 20 | 20 |
| AcK309 | Abz-SKGFGGK(Ac)YGVQKD-NH_2_ | 20 | 2 |

**Table S2:** Primers used for generation of site-specifically acetylated cortactin variants. Mutations introducing the suppressor TAG codon at desired positions in the cortactin gene are shown in bold.

|  | primer sequence (from 5´ to 3´) | |
| --- | --- | --- |
| K87TAG | fw: | caagtcatggttatggtggt**tag**tttggtgttgaacaggat |
|  | rv: | atcctgttcaacaccaaa**cta**accaccataaccatgacttg |
| K124TAG | fw: | ttcgtggttttggcgga**tag**tttggcgttcagatg |
|  | rv: | catctgaacgccaaa**cta**tccgccaaaaccacgaa |
| K161TAG | fw: | aagcggttttggtggc**tag**tacggtgttcaggc |
|  | rv: | gcctgaacaccgta**cta**gccaccaaaaccgctt |
| K198TAG | fw: | cagcaaaggctttggcggt**tag**tatggtatcgataaaga |
|  | rv: | tctttatcgataccata**cta**accgccaaagcctttgctg |
| K235TAG | fw: | gaaaggcttcggaggc**tag**tttggagtgcagac |
|  | rv: | gtctgcactccaaa**cta**gcctccgaagcctttc |
| K272TAG | fw: | ccggctttggtggg**tag**ttcggcgtgcag |
|  | rv: | ctgcacgccgaa**cta**cccaccaaagccgg |
| K309TAG | fw: | ctatagtaaaggtttcggtggt**tag**tatggtgtgcagaaag |
|  | rv: | ctttctgcacaccata**cta**accaccgaaacctttactatag |


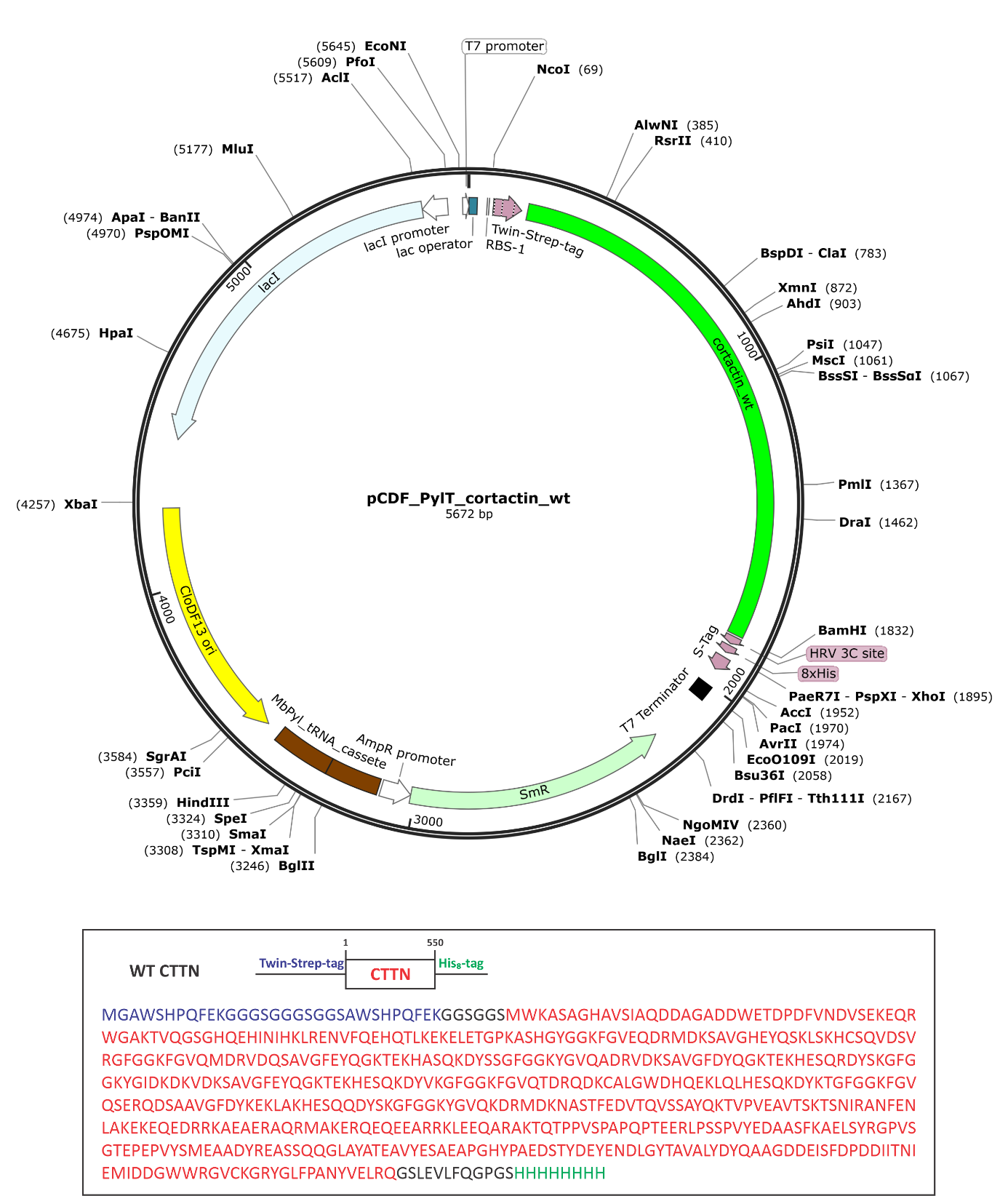


**Figure S1:** The plasmid map and the amino acid sequence of the wild-type cortactin expression construct. The cortactin sequence (human isoform 1, Uniprot accession Q14247-1) is in red, N-terminal Twin-Strep-tag in blue and C-terminal His-tag in green.

**AcK87 AcK124**


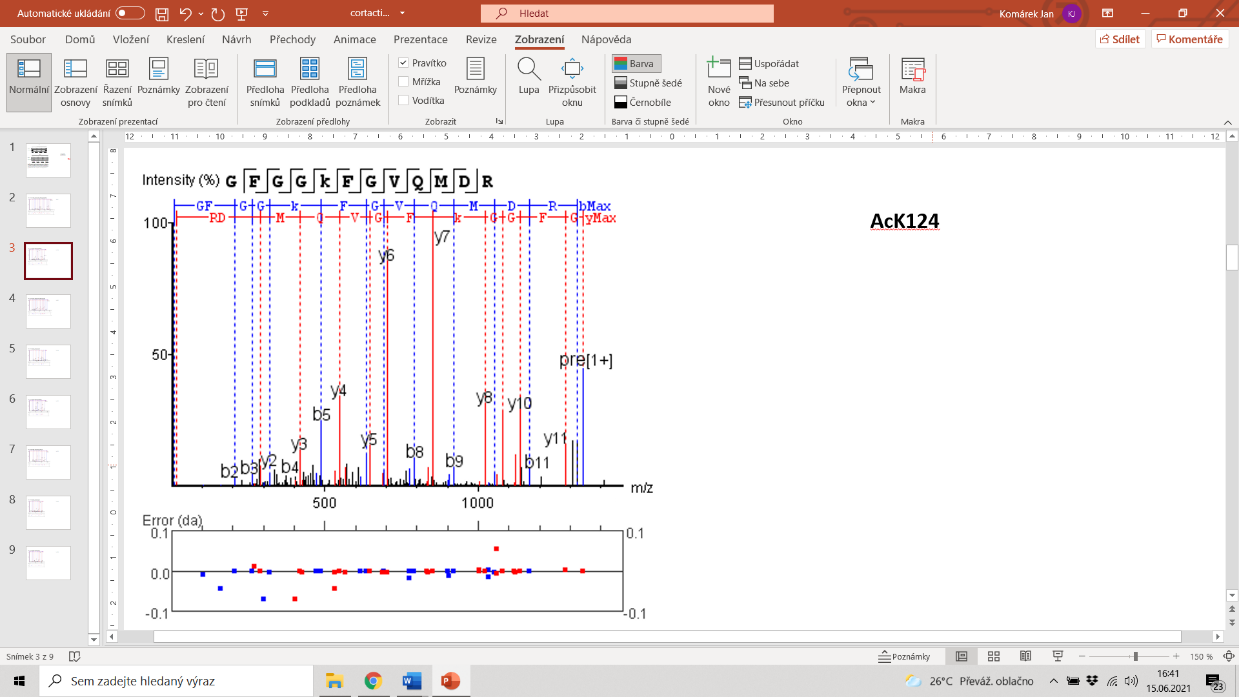

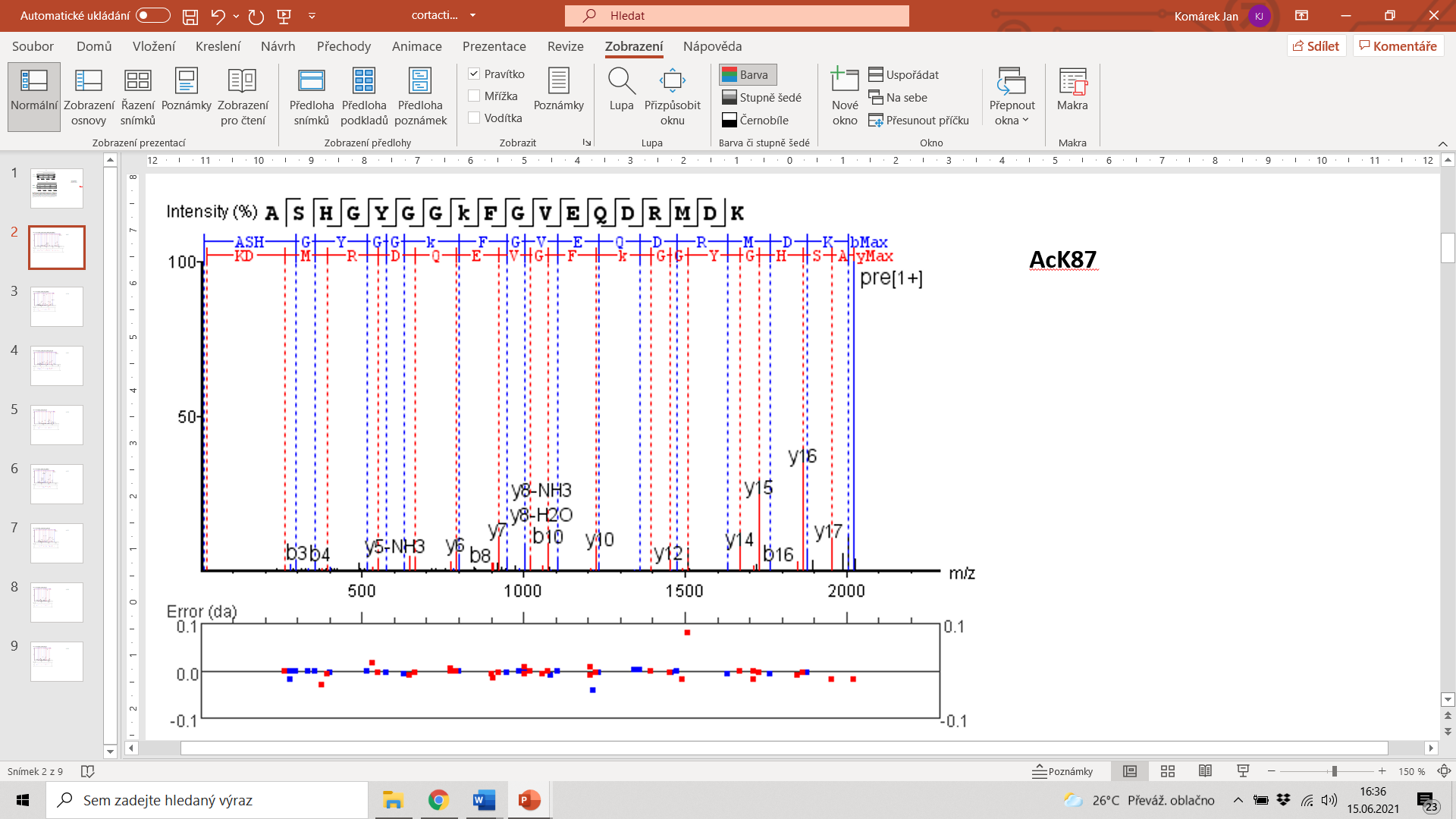


**AcK161 AcK198**


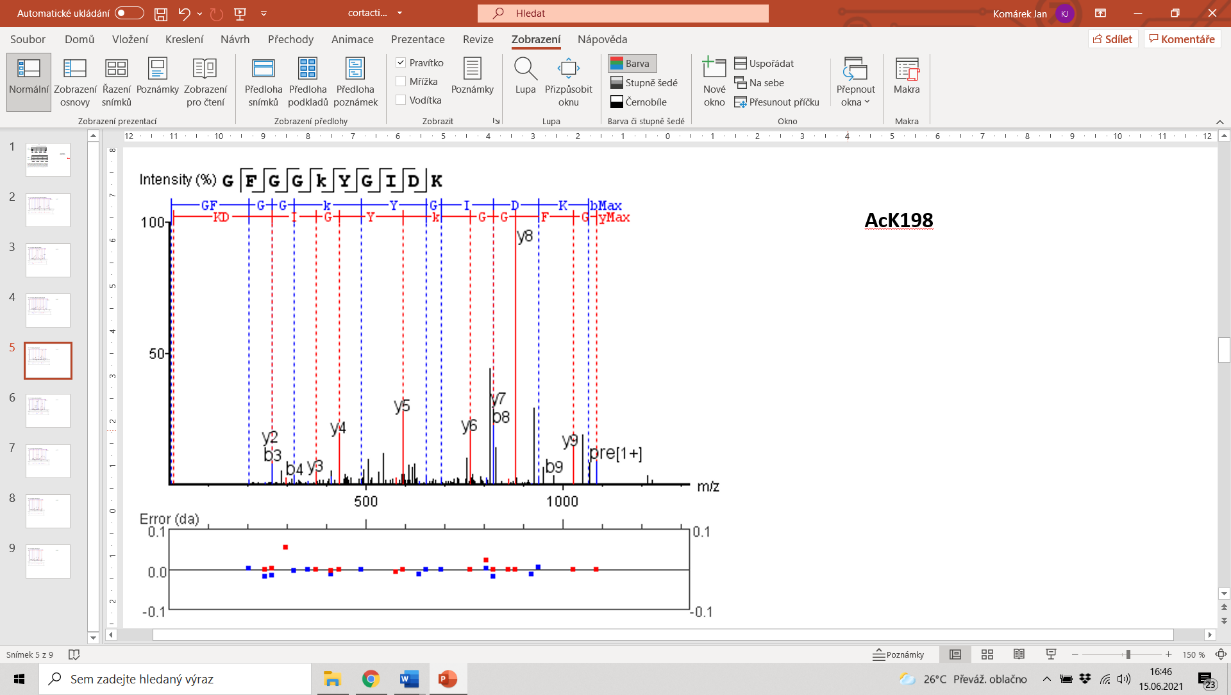

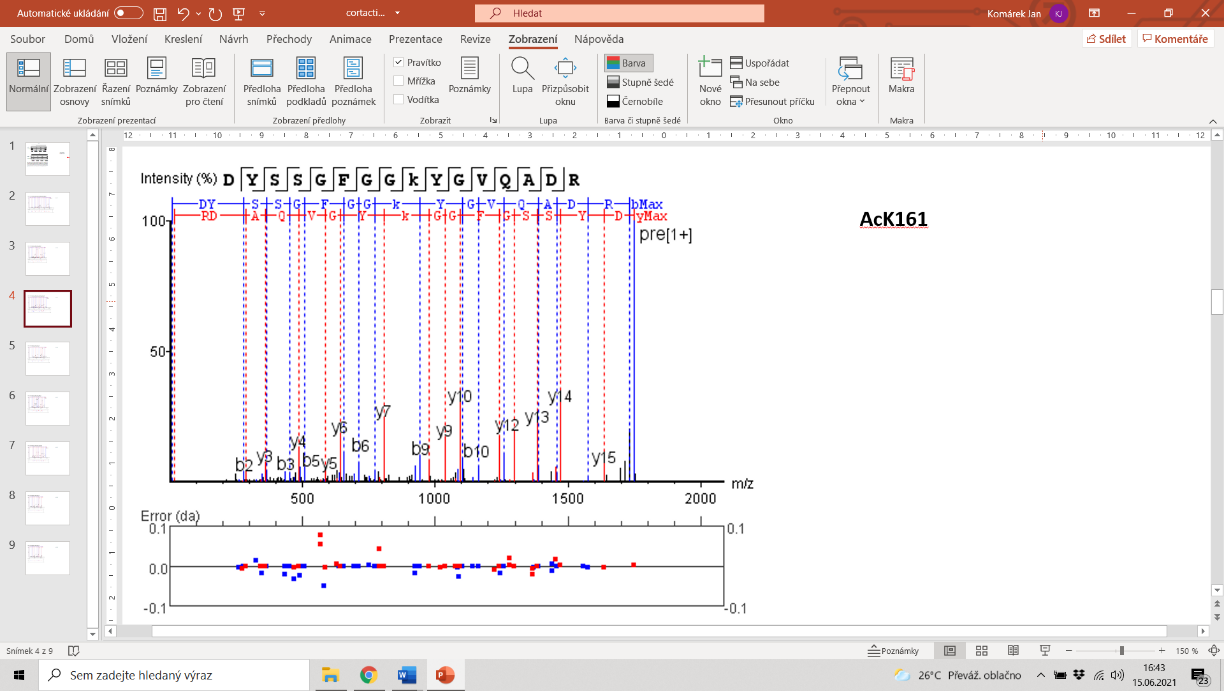


**AcK235 AcK272**


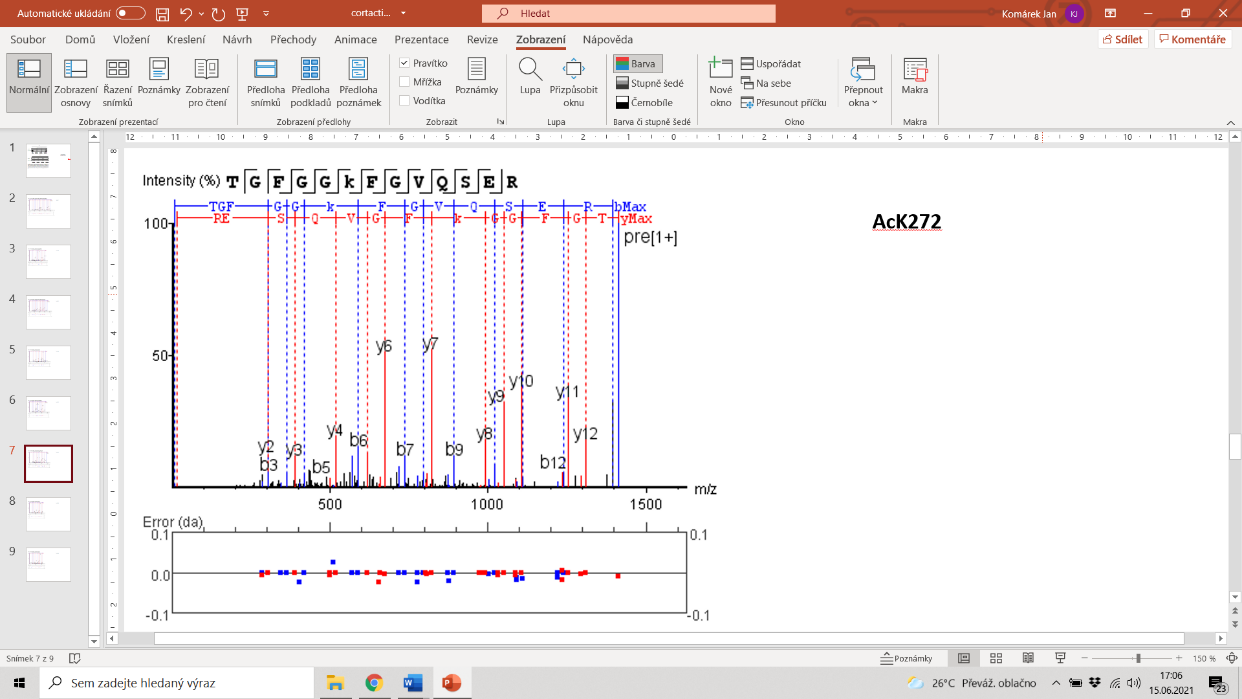

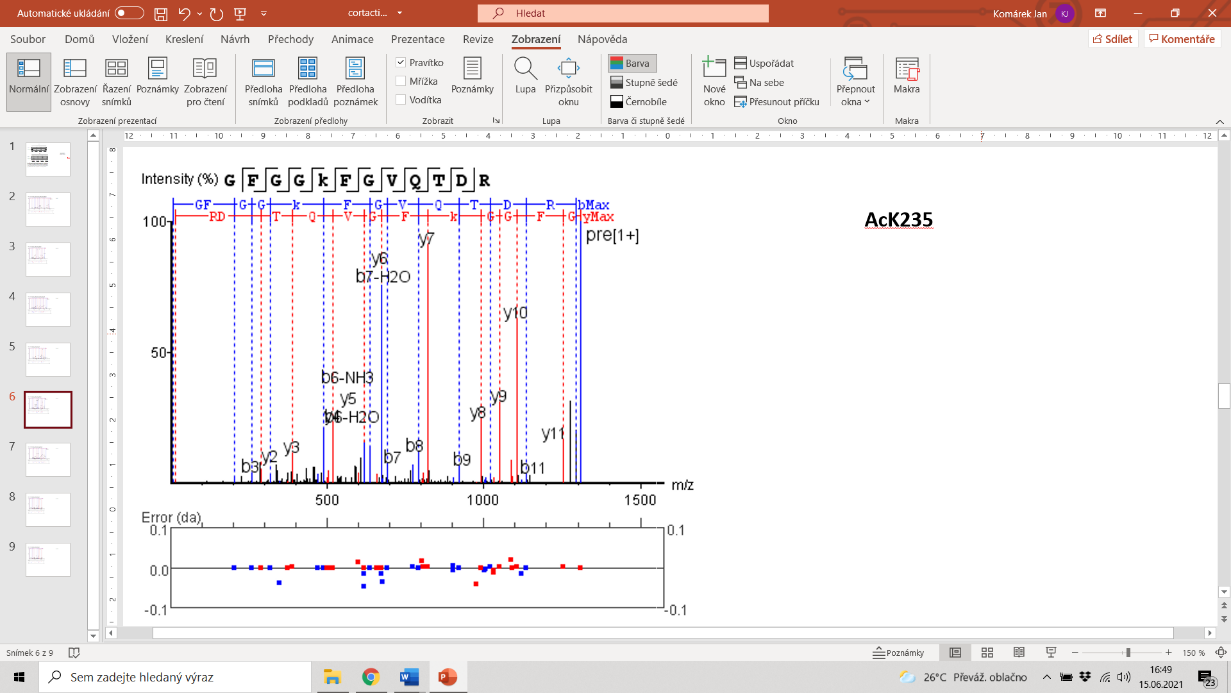


**AcK309**


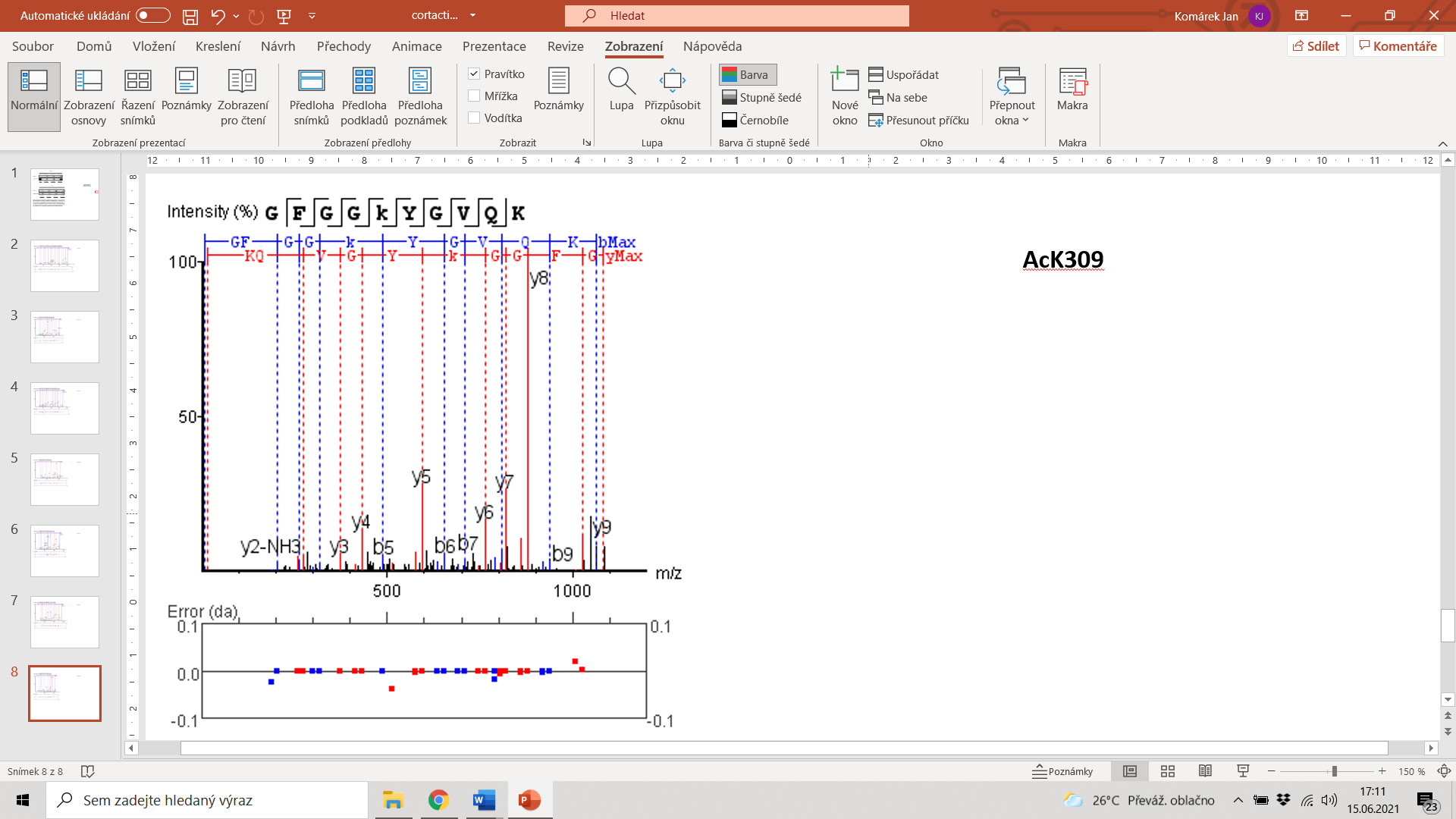


**Figure S2:** MS/MS analysis of tryptic cortactin peptides with the expected sites of acetyl-lysine incorporation. The data confirm the presence of acetyl-lysine at desired positions in all AcK-CTTN constructs. Each panel shows the fragmentation data with the sequence of the tryptic peptide (top), and the graph of error of each ion identified (bottom). The position of the acetyl-lysine is marked as K*. The fragmentation data were analyzed in PEAKS Studio software.

**
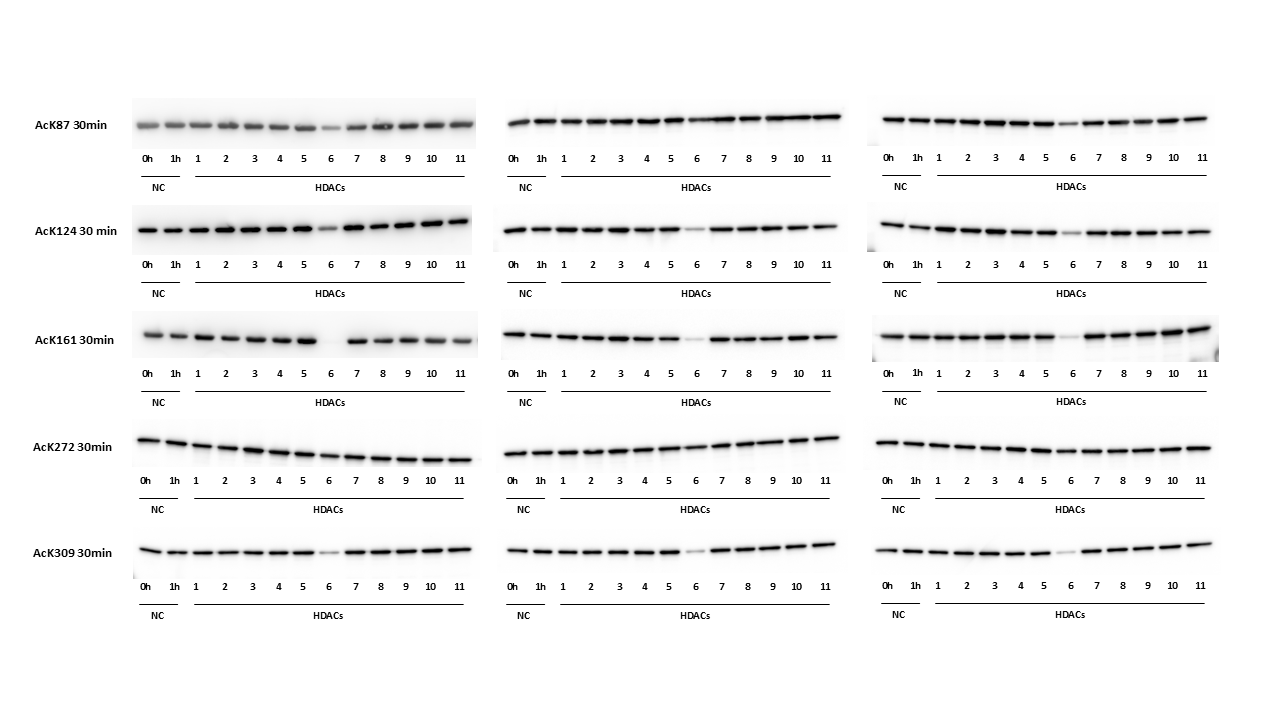
**

**
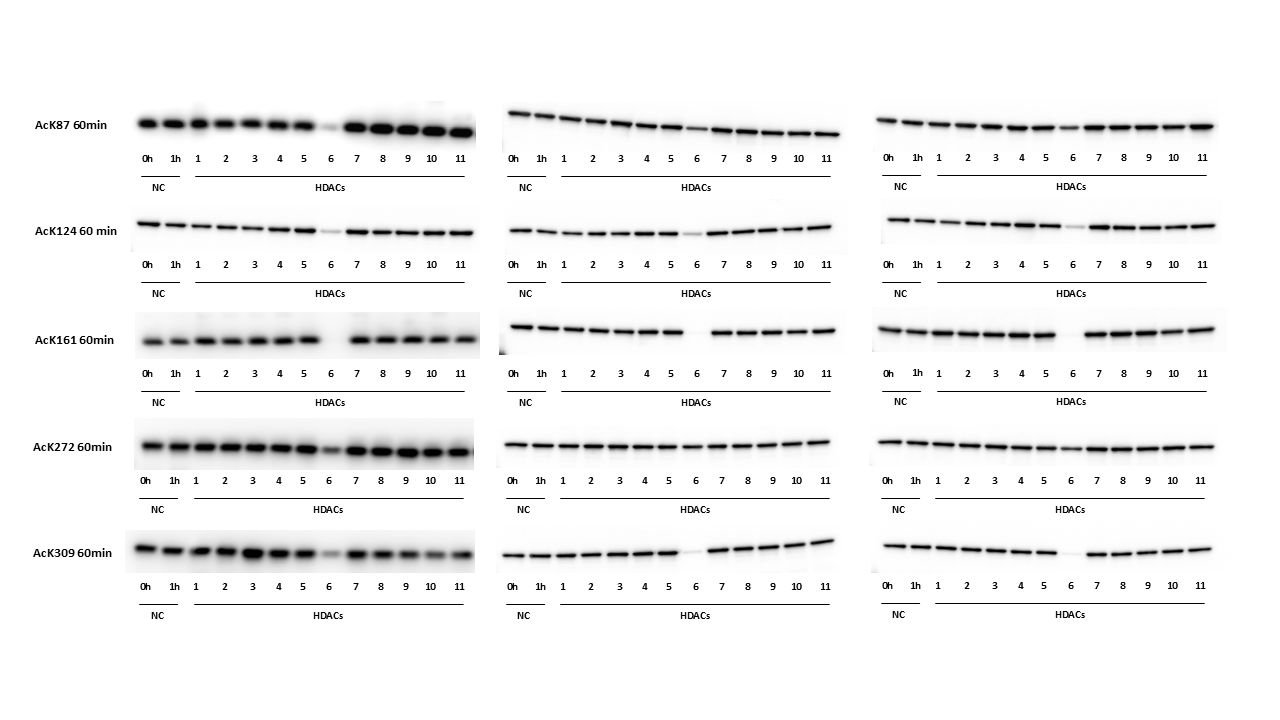
**

**Figure S3:** Replicates of Western blots of deacetylation reactions of AcK-CTTN variants by human HDACs 1–11 (Fig. 2 of the main text). AcK-CTTNs (0.5 μM) were incubated with purified HDACs (50 nM) at 37 °C. Reaction aliquots (30 and 60 minutes) were separated by SDS-PAGE, electrotransferred to a PVDF membrane, and probed with the α-AcK antibody with chemiluminescence detection. Negative controls (NC, no enzyme) at 0 and 60 minutes showed no spontaneous deacetylation. Among all HDACs tested, only HDAC6 induced a marked decrease in AcK-CTTN acetylation signal.

**
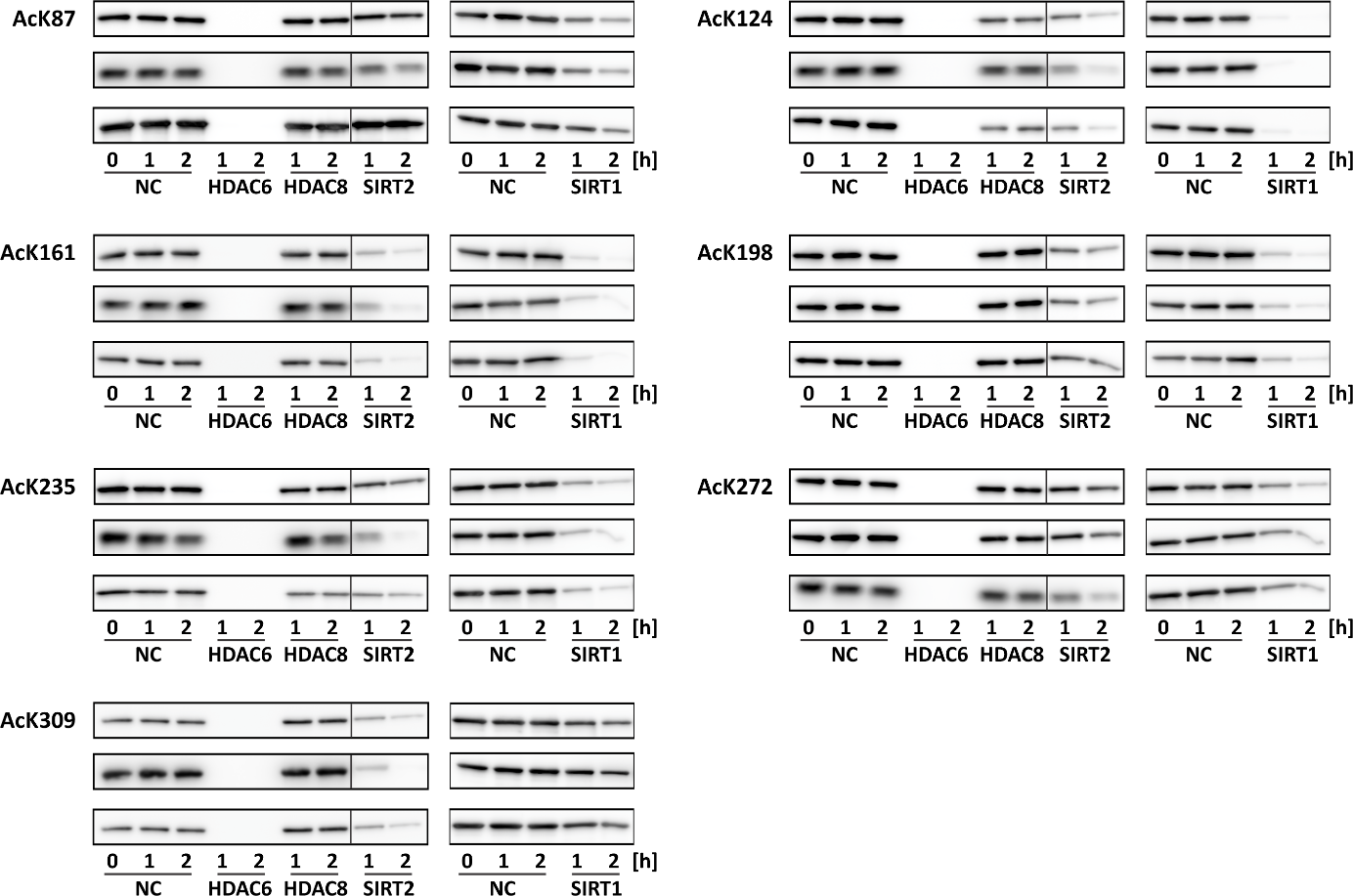
**

**Figure S4:** Replicates of Western blots of deacetylation reactions of AcK-CTTN variants by human HDAC6, HDAC8, SIRT1, and SIRT2 (Fig. 3 of the main text). Western blot analysis of AcK-CTTNs incubated with HDAC6, HDAC8, SIRT1, or SIRT2 at 37 °C for 60 and 120 mins. Reaction aliquots were separated by SDS-PAGE, electrotransferred to a PVDF membrane, and probed with the α-AcK antibody with chemiluminescence detection.

**A**

**
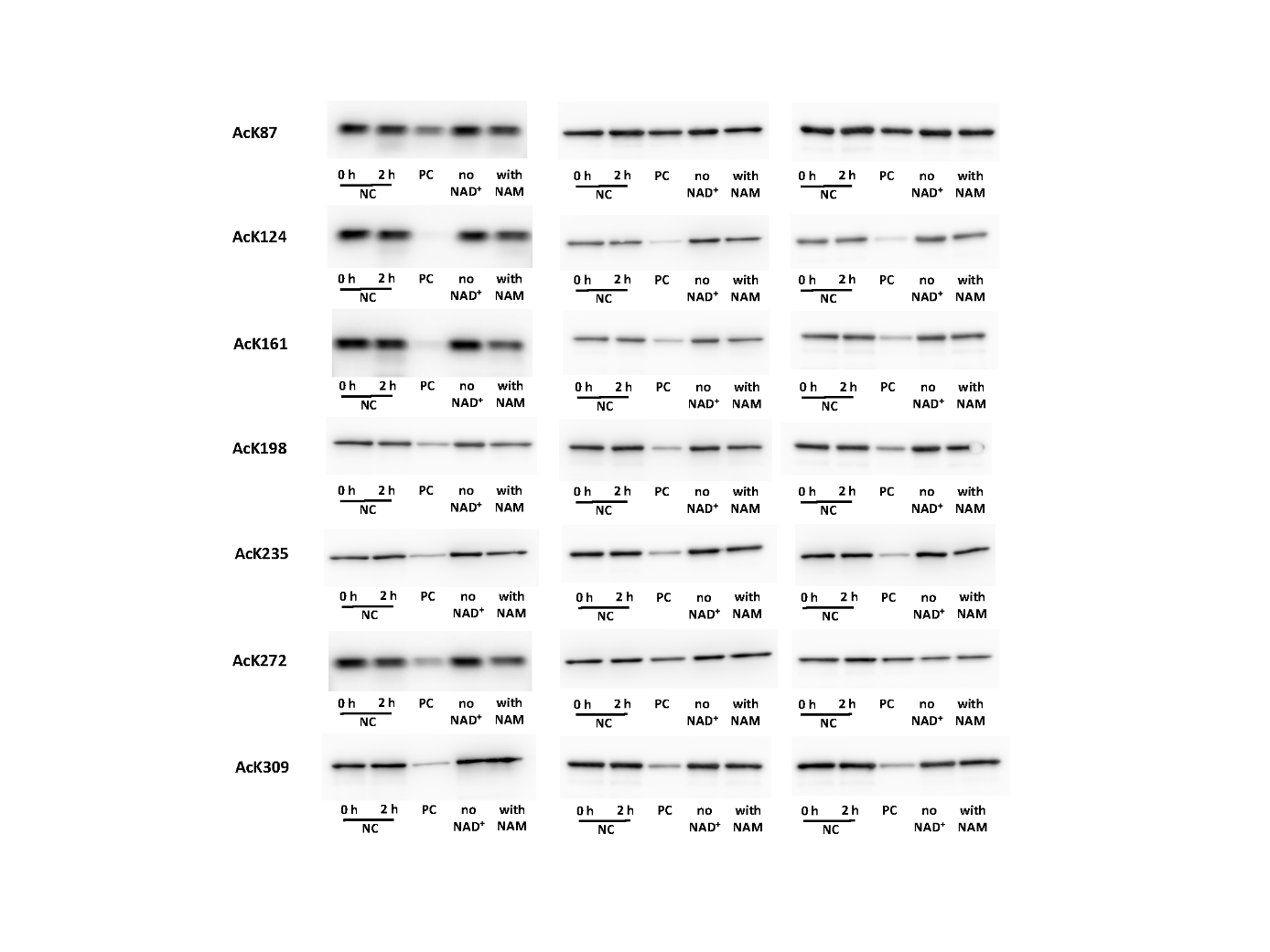
B**

**Figure S5:** Replicates of Western blots of deacetylation reactions by human SIRT2 (Fig. 4 of the main text). AcK-CTTNs variants were incubated with human SIRT2 at 37 °C for 60 (A) and 120 mins (B). Reaction aliquots were separated by SDS-PAGE, electrotransferred to a PVDF membrane, and probed with the α-AcK antibody with chemiluminescence detection. **A:** Concentration-dependent deacetylation of AcK124-CTTN and AcK272-CTTN by SIRT2. WB analysis was performed as described above with SIRT2 concentrations, 0.04, 0.2, and 0.4 μM. **B:** The effect of NAD^+^ and NAM (10 mM concentrations) on SIRT2-catalyzed deacetylation of AcK-CTTN variants.

**
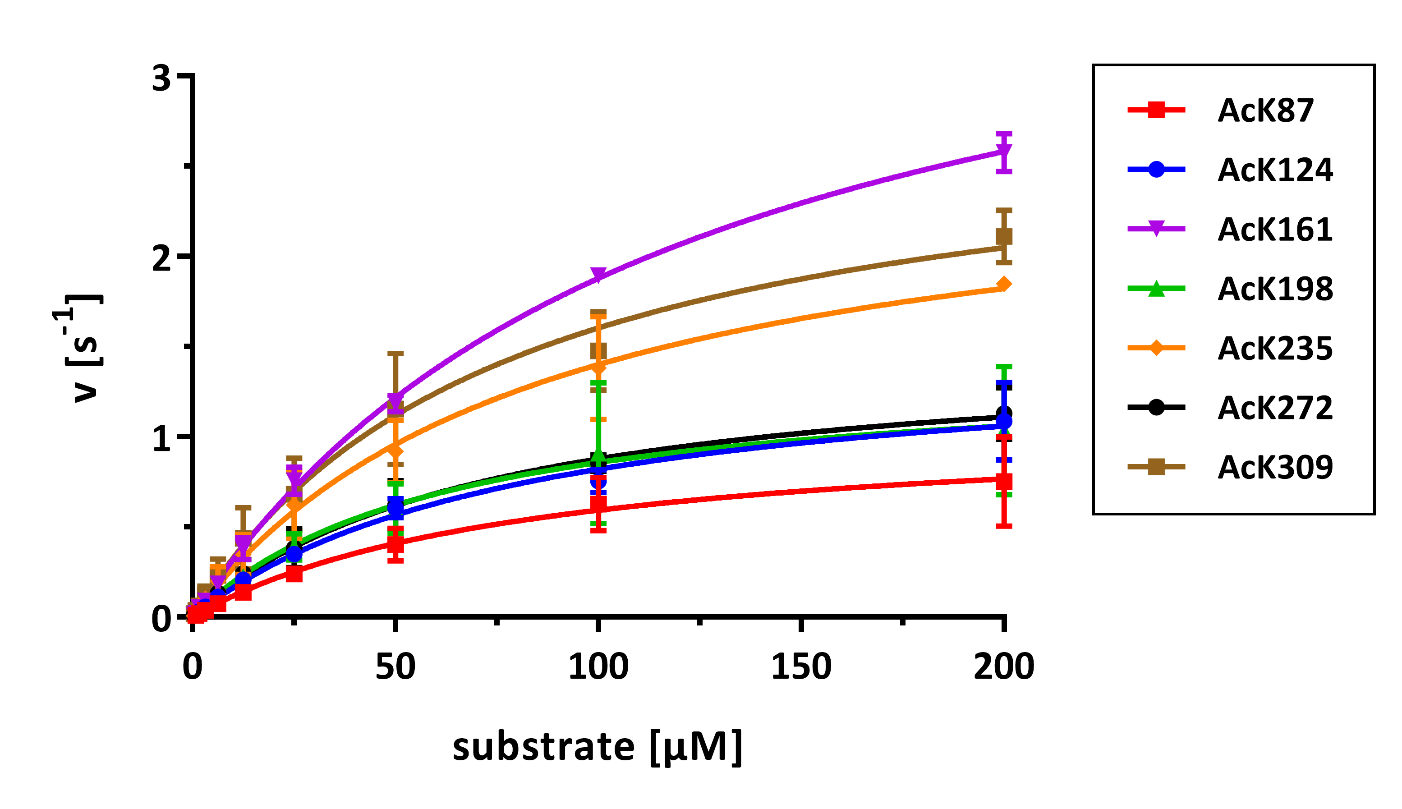
**

**Figure S6:** The Michaelis-Menten plot of deacetylation of AcK-CTTN-derived peptides by wild-type HDAC6. The velocities were determined in 50 mM HEPES, 140 mM NaCl, 10 mM KCl, 0.1% bovine serum albumin (BSA), and 1 mM TCEP (pH 7.4) at 37 °C for 30 minutes. Each analysis was done in triplicate, and error bars indicate the mean ± S.D. from three independent experiments. Kinetic data are reported in Fig. 5A.


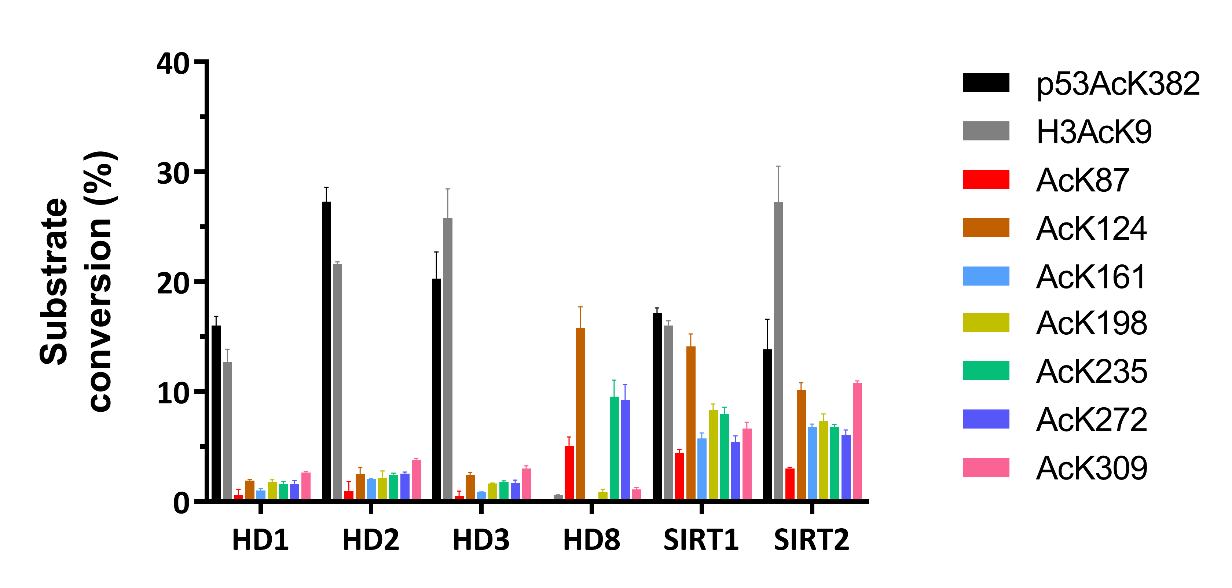


**Figure S7:** **Deacetylation of substrate-derived peptides by selected HDACs.** 20 µM peptides were incubated with selected HDACs at 37°C for 30 mins and the deacetylation levels determined by an RP-HPLC-based assay. HDAC concentrations: HDAC1 (125 nM), HDAC2 (2000 nM), HDAC3 (0.8 nM), HDAC8 (50 nM), Sirt1 (10 nM), and Sirt2 (50 nM). HDACs 1, 2, 3, and Sirt2 preferentially deacetylate H3K9 and p53K382 peptides, while HDAC8 shows preference for selected AcK-CTTN peptides. Low deacetylation activity (~1-3%) on AcK-CTTN peptides is observed for HDAC1, 2, and 3 under these assay conditions. Bar graphs represent the mean ± S.D. from three independent experiments.


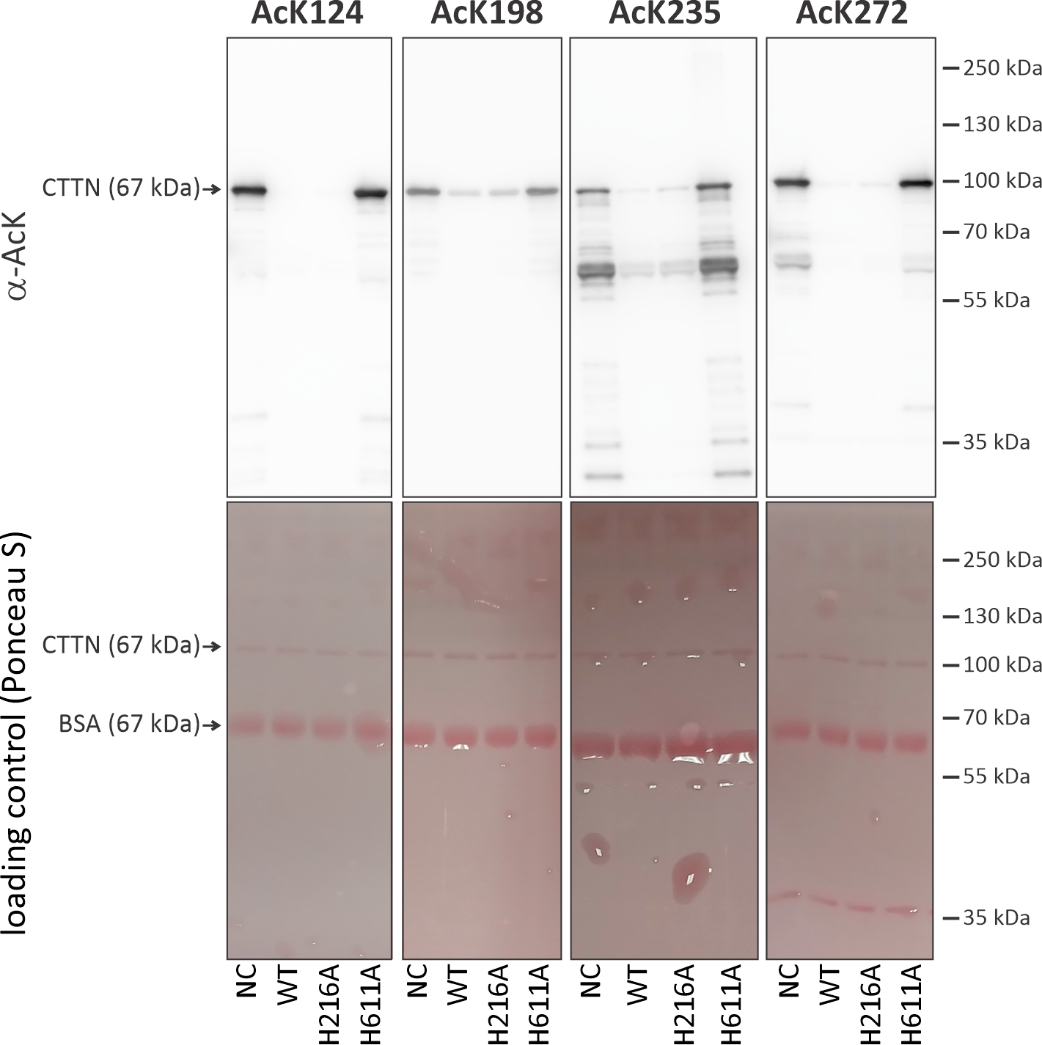


**Figure S8:** Uncropped representative Western blots of deacetylation reactions of AcK-CTTN by HDAC6 variants. Membranes were stained by anti-AcK antibody (upper panel) and Ponceau S (lower panel). AcK-CTTN proteins (0.5 µM) were incubated with 250 nM HDAC6 variants at 37 °C for 2 h. Acetylation levels were assessed by immunoblotting using the α-AcK antibody (n = 3); Ponceau S staining served as a loading control. All tested CTTN variants (AcK124, AcK198, AcK235 and AcK272) were deacetylated by wild-type HDAC6 and the H216A mutant, while the H611A mutant showed no activity. NC - negative control (no HDAC).


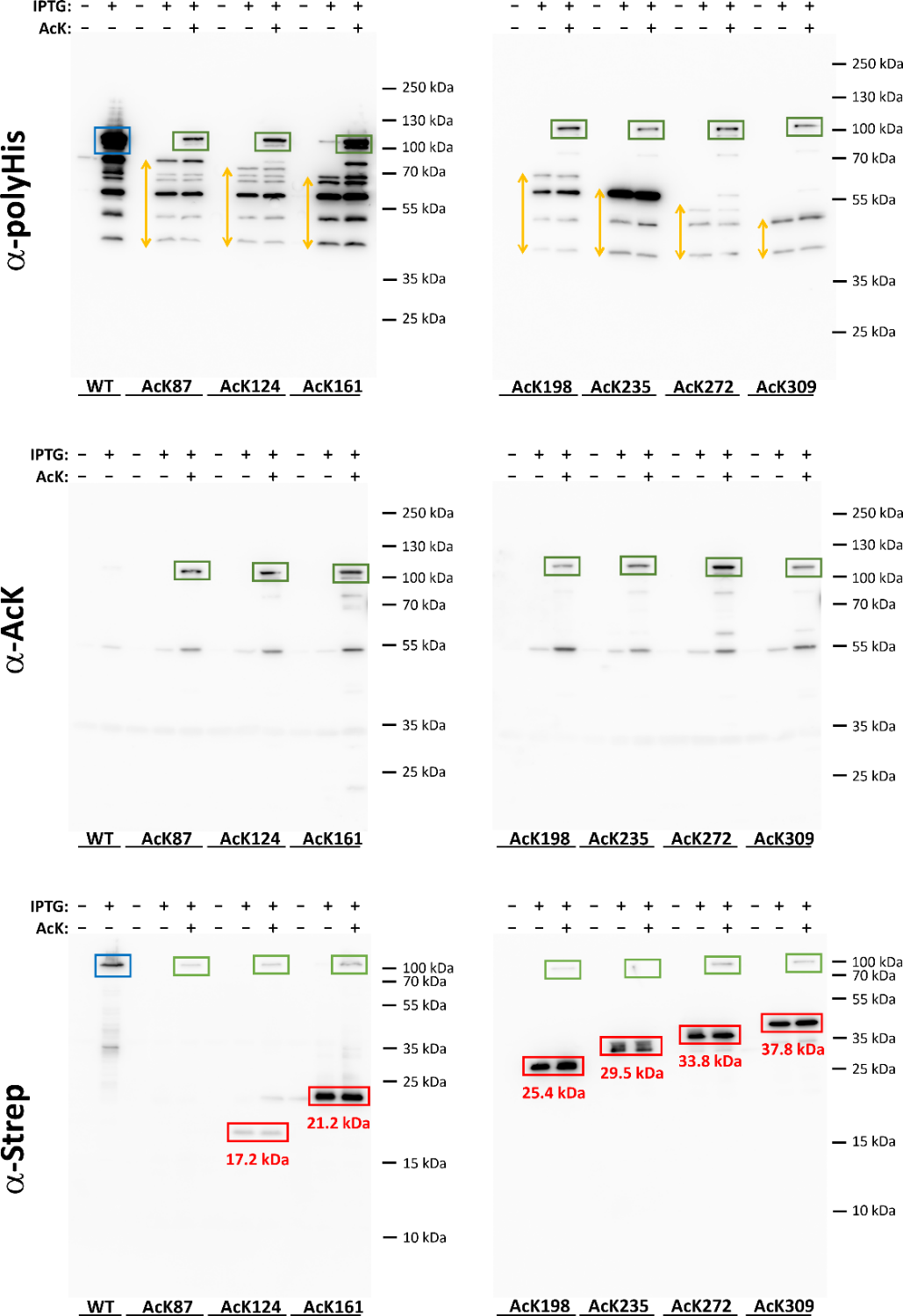


**Figure S9:** Uncropped representative Western blots of expression of wild-type and AcK-CTTN variants in *E. coli*.

Western blot analysis of cell lysates demonstrates successful incorporation of N-acetyl-lysine (AcK) at defined positions in response to amber (TAG) stop codons, and the production of full-length AcK-CTTN only in the presence of externally supplied AcK (upper and middle panels). The upper panel (α-polyHisantibody) also shows partial CTTN degradation and the presence of N-terminally truncated CTTN variants. The lower panel (α-Strep antibody) reveals the presence of C-terminally truncated AcK-CTTN variants resulting from premature termination of translation at the TAG sites. The experiments were repeated three times.

Cell lysates were prepared by disrupting the bacterial cells in 4 M urea. The proteins were separated by SDS-PAGE (10% or 12% gels), transferred to PVDF membrane and stained with α-polyHis, α-AcK and α-Strep antibodies. Wild-type CTTN, AcK-CTTNs, and C-terminally truncated AcK-CTTNs are marked by blue, green and red boxes, respectively. The N-terminally truncated CTTN fragments and/or their degradation products are indicated by orange arrows.

**
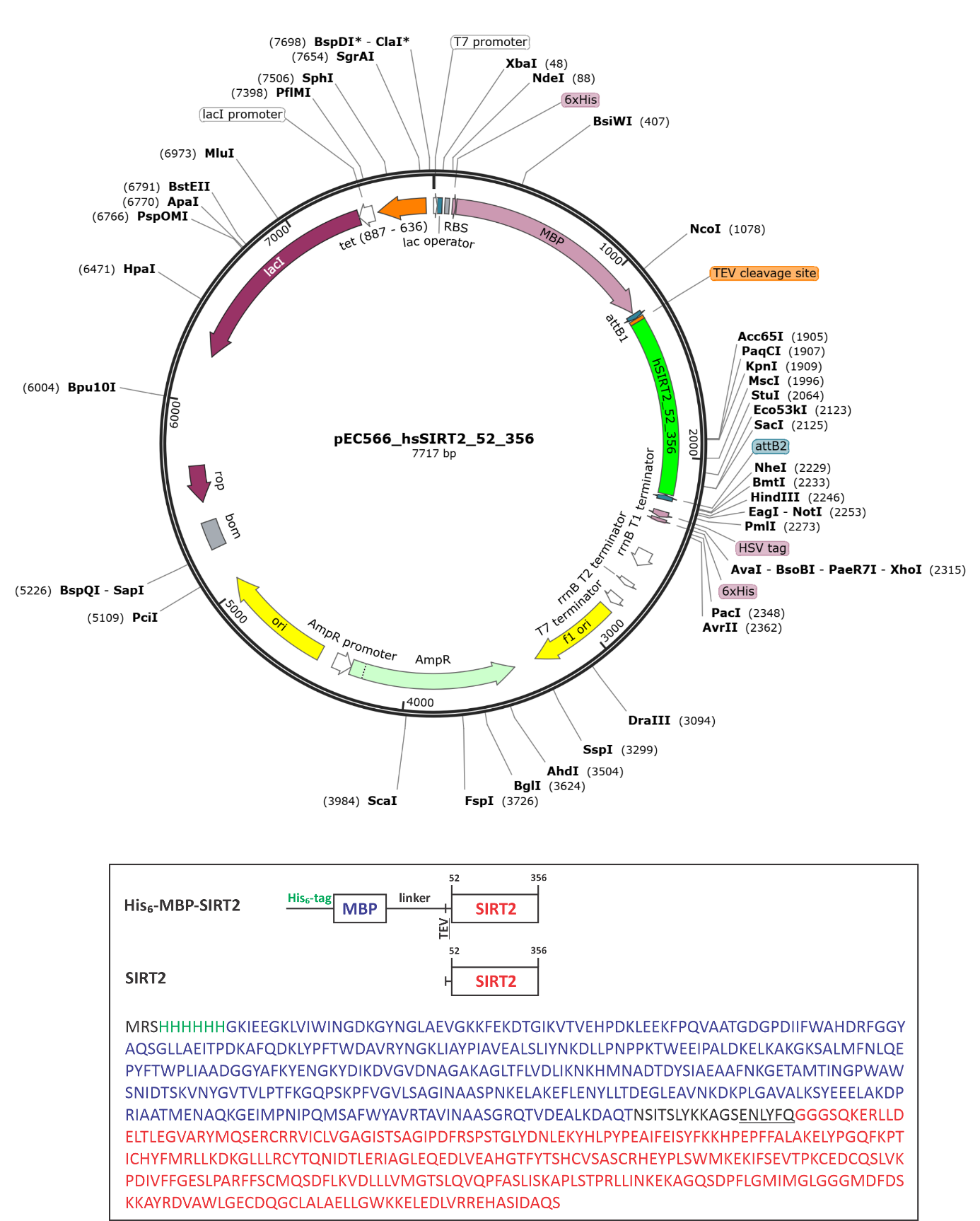
**

**Figure S10:** The plasmid map and the amino acid sequence of the SIRT2 expression construct. The human SIRT2 sequence (amino acids 52-356; Uniprot Q8IXJ6) in red, the N-terminal His_6_-MBP tag (green-blue). The TEV site sequence is underlined. During purification, the N-terminal His_6_-MBP tag was cleaved off by a TEV protease and subsequently removed by Ni-NTA chromatography.


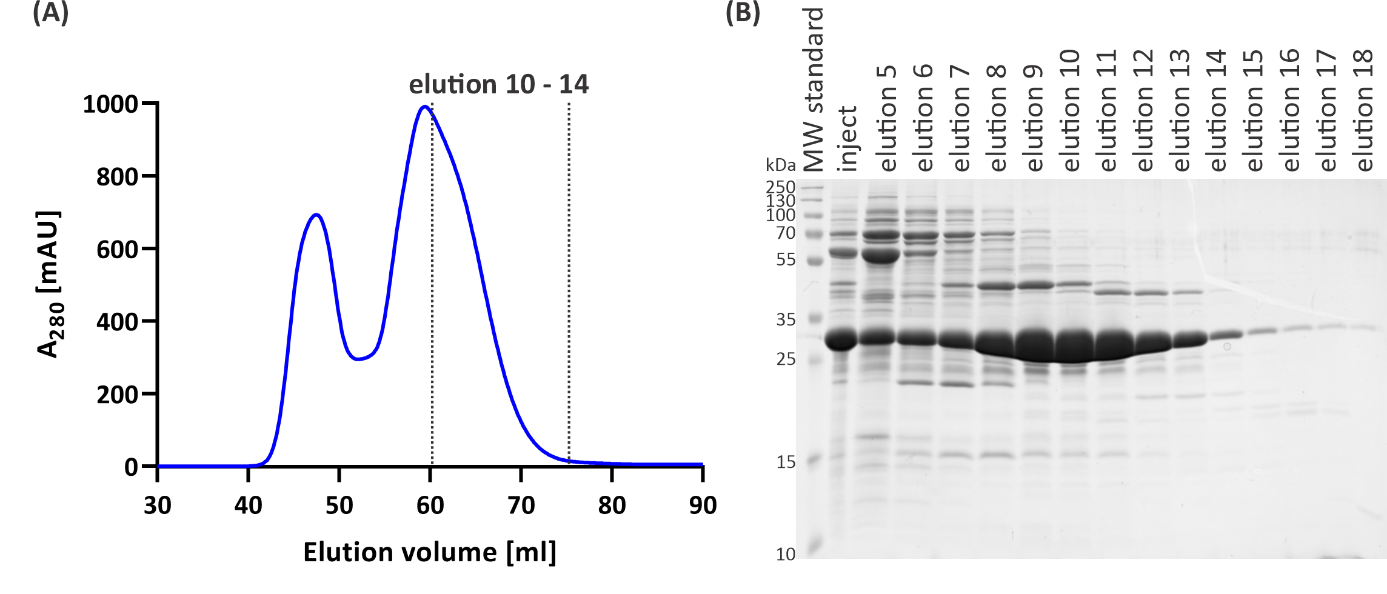


**Figure S11:** Purification of human SIRT2. The final purification step comprised size exclusion chromatography (A) using a Superdex 75 16/600 column (Cytiva) equilibrated in 20 mM Tris-HCl, 150 mM NaCl, 10 mM KCl, pH 8. SDS-PAGE analysis of elution fractions is shown in panel B. Purified SIRT2 (fractions 10-14) was concentrated, flash-frozen in liquid nitrogen and stored at -80 °C.


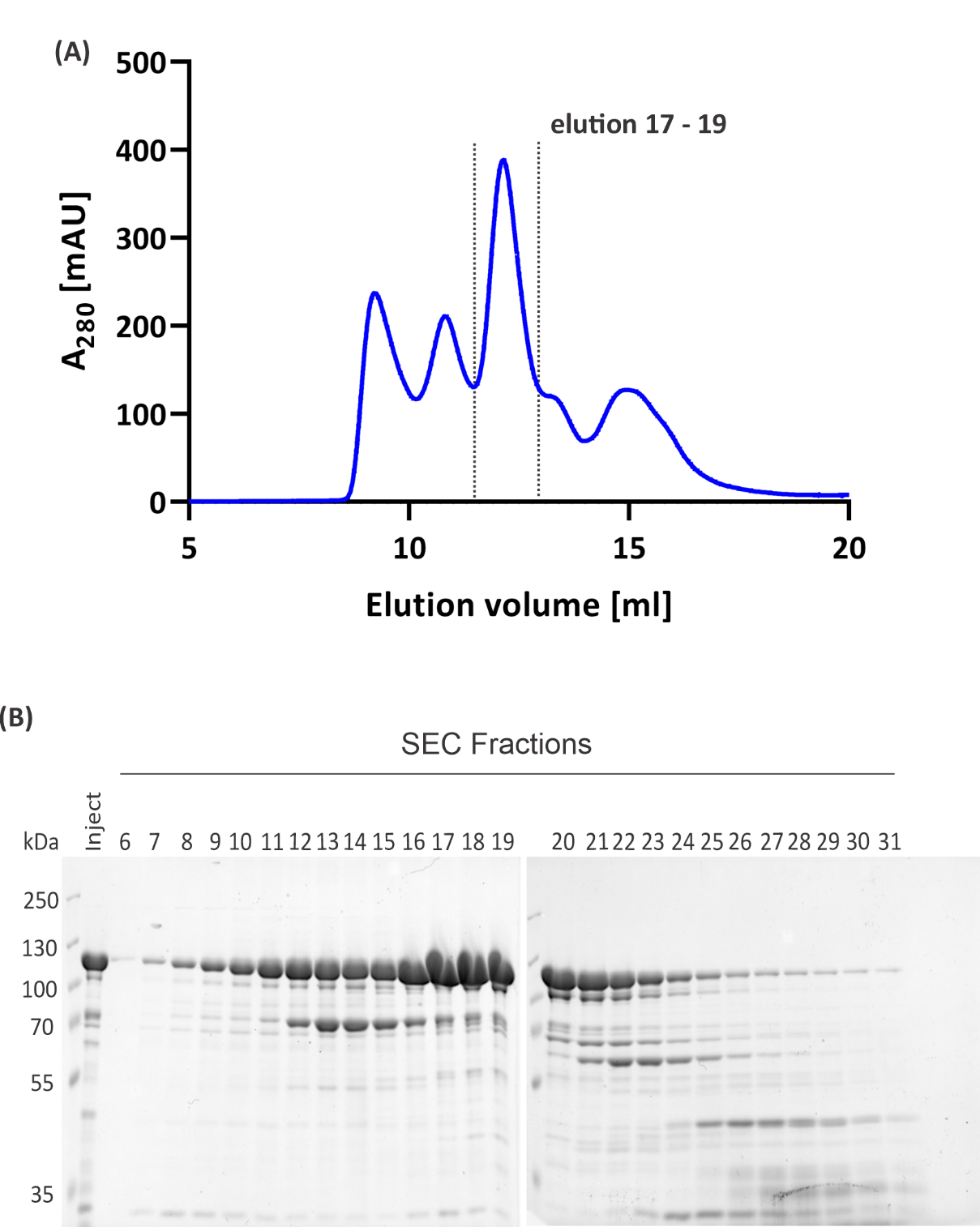


**Figure S12:** Purification of human His_6_-SUMO-SIRT1 fusion. The final purification step comprised size exclusion chromatography (A) HiLoad Superdex 200 pg 10/300 column (Cytiva) equilibrated in 25 mM Tris–HCl, 300 mM NaCl, 1.5 mM DTT, pH 7.6. SDS-PAGE analysis of elution fractions is shown in panel B. Purified SIRT1 (fractions 17-19) was concentrated, flash-frozen in liquid nitrogen and stored at -80 °C.

**
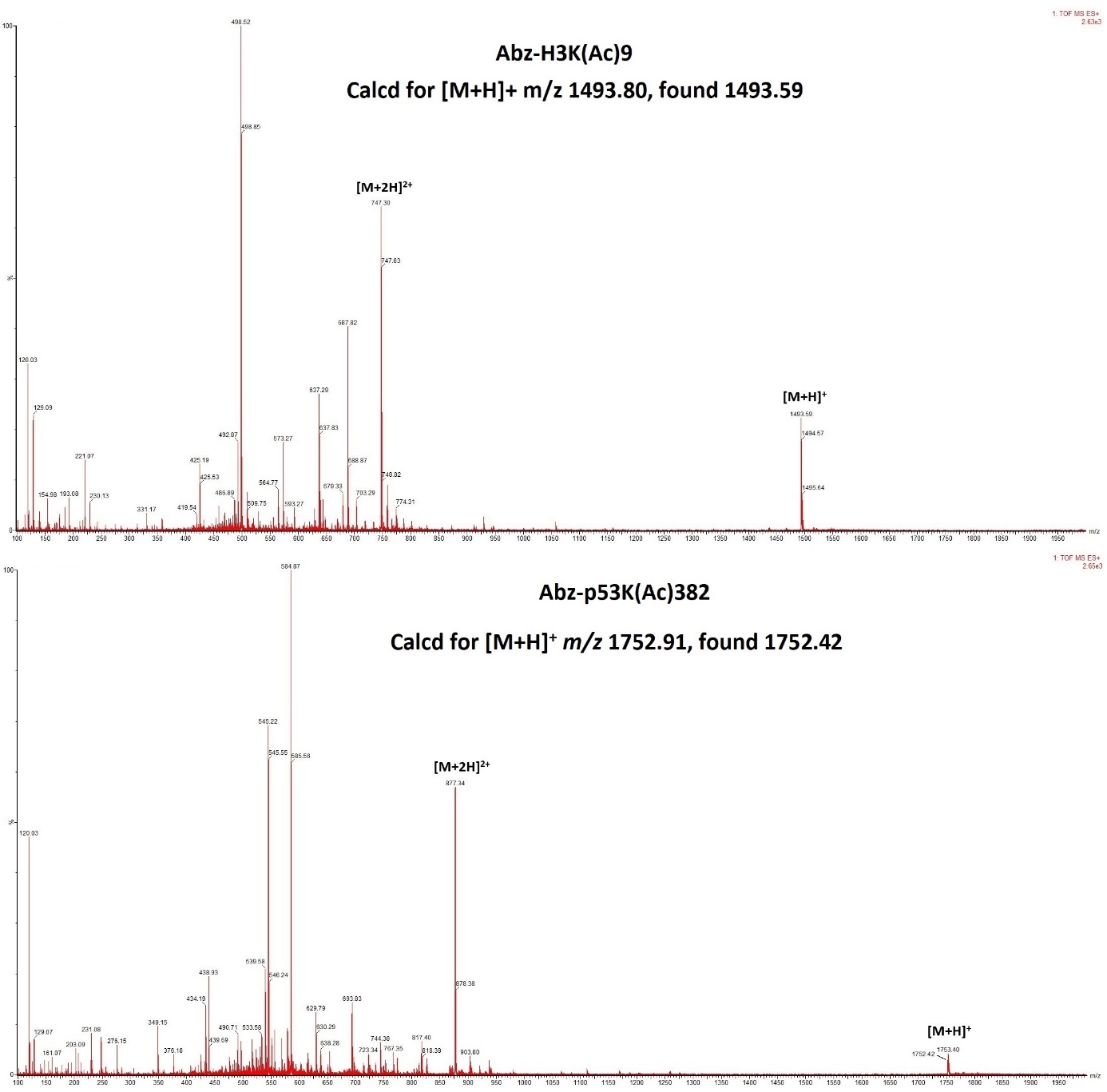
**

**Figure S13: QTOF-MS (ESI+) spectra of H3K9 and p53AcK382 peptides as class I HDAC substrates**. QTOF spectra were measured using the Waters Q-Tof Premier (Milford, MA, USA) mass spectrometer. Data were analyzed using Waters MassLynx software.
